# Screening and Comprehensive Evaluation of Drought Resistance in Cotton Germplasm Resources at the Germination Stage

**DOI:** 10.3390/plants14142191

**Published:** 2025-07-15

**Authors:** Yan Wang, Qian Huang, Li Liu, Hang Li, Xuwen Wang, Aijun Si, Yu Yu

**Affiliations:** 1 The Key Laboratory of Oasis Eco-Agriculture, Agriculture College, Shihezi University, Shihezi 832003, China; wyxlb28@163.com; 2 Cotton Research Institute, Xinjiang Academy of Agricultural and Reclamation Science/Northwest Inland Region Key Laboratory of Cotton Biology and Genetic Breeding, Shihezi 832000, China; hq10200102@163.com (Q.H.); cottonliuli@sina.com (L.L.); lhup1997@163.com (H.L.); wxw629@163.com (X.W.)

**Keywords:** cotton, germination, drought stress, comprehensive evaluation, phenotypic trait

## Abstract

Drought stress has a significant impact on cotton growth, development, and productivity. This study conducted drought stress treatment and normal water treatment (control group) on 502 cotton accessions and analyzed data on eight phenotypic traits closely related to drought stress tolerance. The results showed that all indicators changed significantly under drought stress conditions compared to the control group, with varying degrees of response among different indicators. To comprehensively evaluate the drought resistance of cotton during the germination period, the values of drought resistance comprehensive evaluation (D-value), weight drought resistance coefficient (WDC-value), and comprehensive drought resistance coefficient (CDC-value) were calculated based on membership function analysis and principal component analysis. Cluster analysis based on the D-value divided the germplasm into five drought-resistant grades, followed by the selection of one extreme material, each from the strongly drought-resistant and strongly drought-sensitive groups. An evaluation model was established using stepwise regression analysis, including the following effective indicators: Relative Fresh Weight (RFW), Relative Hypocotyl Length (RHL), Relative Seeds Water Absorption Rate (RAR), Relative Germination Rate (RGR), Relative Germination Potential (RGP), and Relative Drought Tolerance Index (RDT). The validation of the D-value prediction model based on the Best Linear Unbiased Prediction (BLUP) showed that the results obtained from two independent biological replicates were highly consistent. The comprehensive evaluation system and screening indicators established in this study provide a reliable method for identifying drought tolerance during the germination period.

## 1. Introduction

Cotton belongs to the genus *Gossypium* of the family Malvaceae and is the world’s most important fiber crop and cash crop [1,2,3], accounting for approximately 35% of global fiber production [4]. As the world’s largest producer and exporter of textiles, China plays a key role in promoting international trade and driving global consumption and economic growth. The expansion of cotton monoculture reduces soil biodiversity and promotes pathogen accumulation, degrading soil health and long-term land productivity. These effects intensify the “cotton–grain competition” [5] in arid regions, where cotton’s high irrigation demand during germination further strains limited grain farmland. Enhancing germination-stage drought tolerance through breeding could reduce cotton’s reliance on fertile, well-watered land, enabling more sustainable coexistence with food crops. However, the mechanisms related to cotton’s drought tolerance during the germination period remain unclear, and the identification and screening of drought tolerance in germplasm resources have become critical tasks before elucidating these mechanisms.

Drought is one of the major adversity factors in nature [6,7] and the most significant environmental factor affecting agricultural production [8,9]. It is characterized by prolonged water deficiency, which severely hampers plant growth and development [10], leading to significant yield loss or even crop failure [11,12,13,14]. Severe drought stress can cause slowed plant development, thus affecting plant morphology and physiology [15,16,17]. Rising global temperatures and freshwater [18] have contributed to declining crop yields [19,20]. It is projected that the global land area affected by drought will nearly double by the end of the 21st century [21]. Hence, many agricultural researchers are now focusing on resolving drought stress tolerance in crops to cultivate superior drought-tolerant germplasm [22,23], thereby enhancing crop yield and quality.

Assessing crop drought tolerance is complex due to stage-environment interactions, necessitating multi-indicator approaches that combine physiological, biochemical, and morphological traits [24]. The germination stage shows particular drought sensitivity, making it ideal for rapid drought-resistant variety screening [25]. While germination-stage drought screening has been successfully applied across crops, methodologies vary significantly. Jia et al. conducted drought tolerance screening on 264 soybean accessions and successfully integrated six indicators, including GR, GE, and GI, to identify 17 strongly drought-tolerant accessions [26]. Bao et al. collected and analyzed 24 traits of winter wheat and ultimately screened out five drought-resistant varieties based on 10 key indicators [27]. Wu et al. combined GR and RL indices to identify one strongly drought-tolerant and seventeen sensitive *Sorghum* varieties [28]. Regrettably, current research on drought tolerance in cotton has primarily focused on later growth stages, such as seedling establishment and boll development, while evaluation systems for drought responses during the germination stage remain inadequately developed.

In recent years, as analytical methods have advanced significantly, the evaluation of crop stress resistance can no longer be comprehensively assessed through single or even multiple isolated indicators [29]. Consequently, researchers are adopting integrated analytical approaches, including variance analysis, principal component analysis, and fuzzy membership function analysis [30,31,32,33,34,35,36], to achieve more objective, accurate, and scientifically robust screening results [37,38,39].

This study innovatively selected 502 genetically diverse cotton accessions at the germination stage for drought tolerance assessment. An efficient screening system was developed by selecting and integrating multiple excellent evaluation indices, and drought-resistant cotton accessions were successfully identified. These findings facilitate the selection of drought-tolerant germplasm for breeding programs and provide valuable tools for investigating drought-tolerance mechanisms. This work contributes significantly to sustainable cotton cultivation in Xinjiang’s arid regions, with important implications for agricultural productivity and economic development.

## 2. Results

### 2.1. Effect of Drought Stress on Major Agronomic Traits

In this study, we planted 502 cotton accessions in two biological replicates (REP1 and REP2) and performed phenotypic data analysis under normal water (CK) and drought stress (DS) conditions.

The results showed that in REP1, the coefficient of variation (CV) for all traits under CK conditions ranged from 10.46% to 32.79% (Table 1). Compared with the CK treatment, the CV values of all traits increased to a range of 28.16% to 85.60% after DS treatment, showing significant drought inhibition effects. Among them, the decrease in RL was the smallest (24.87%), while the inhibition of HL was the most severe (79.63%).

In REP2, the CV values for all traits under CK conditions ranged from 7.06% to 26.26% (Table 2), while the CV values ranged from 29.30% to 67.46% under DS conditions. Compared with CK, RL showed the smallest reduction (8.33%), while DT was most severely affected (72.93%).

**Figure 1:**
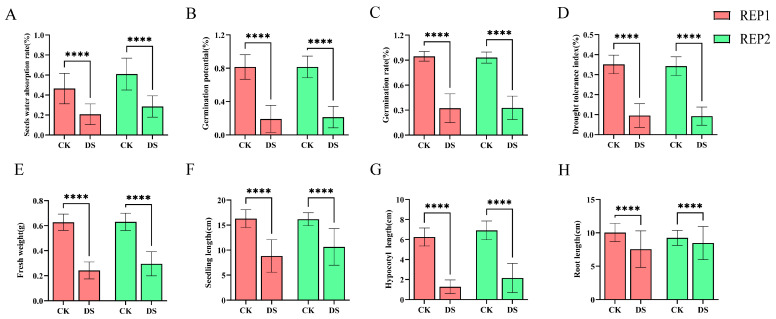
Measurement of eight phenotypic traits in two biological replicates under normal water treatment and drought stress conditions. **** Indicates *p* < 0.0001 (**A**) Seeds water absorption rate; (**B**) germination potential; (**C**) germination rate; (**D**) drought tolerance index; (**E**) fresh weight; (**F**) seedling length; (**G**) hypocotyl length; (**H**) root length.

Combining the experimental results of the two REPs (Figure 1), it was found that GP and HL consistently exhibited significant variation under CK conditions. Under DS conditions, HL showed the most obvious variation and responded most quickly to drought stress, which we speculate may be related to the ABA pathway. RL showed less variation and maintained stable physiological parameters, suggesting that it has stress-resistant buffering properties, which may be related to the accumulation of osmotic regulatory substances. Although different cotton varieties showed similar drought response patterns across replicates, these findings emphasize the necessity of conducting comprehensive multi-trait evaluations rather than single-trait assessments.

### 2.2. Phenotypic Drought Tolerance Coefficients and Correlation Analysis

Under drought stress conditions, eight phenotypic trait indices of two biological replicates from 502 cotton accessions exhibited varying degrees of change, and significant differences were observed in drought tolerance coefficients (DCs) among different accessions.

The CV of DCs ranged from 28.68% to 84.37% in REP1 (Table 3) and from 28.96% to 63.08% in REP2 (Table 4). In both REPs, RFW exhibited the lowest variation, indicating its relative stability. The variation of DC across different indicators further highlights the differential sensitivity of each trait to drought stress.

Further correlation analysis of drought coefficients (DCs) revealed that in REP1, RAR showed significant negative correlations with RGP, RGR, and RDT while being negatively correlated with all other measured traits. Strong positive correlations were observed between RGE, RGR, RDT, RFW, RSL, RHL, and RRL. The strongest correlation was found between RSL and RRL (r = 0.969, *p* < 0.01), while the weakest association was between RGP and RHL (r = 0.453, *p* < 0.01) (Table 5).

In REP2, RAR showed a highly significant negative correlation with all other traits. RGP, RGR, RDT, RFW, RSL, RHL, and RRL again showed strong positive intercorrelations. The RGR and RDT pair exhibited the strongest relationship (r = 0.951, *p* < 0.01), while RGP and RRL demonstrated the weakest correlation (r = 0.541, *p* < 0.01) (Table 6).

These results collectively demonstrate consistent correlation patterns among DC values across replicates. RAR consistently showed inverse relationships with other traits, while other parameters maintained strong positive correlations. This consistency confirms the representativeness of our phenotypic data for comprehensive drought stress tolerance evaluation, supporting the reliability of using these traits for germplasm assessment. The particularly strong RSL and RRL suggest coordinated growth regulation under drought stress, while the variable RAR relationships may indicate its role as an independent stress adaptation mechanism.

### 2.3. Principal Component Analysis

In REP1 (Table 7), PCA identified three major components (λ > 0.848) that collectively explained 89.206% of the total variance. These composite drought tolerance indices (PC1, PC2, PC3) effectively captured the genetic information from all eight original traits. PC1, accounting for 41.799% of the variance (λ = 4.94), showed strong loadings on RSL, RGR, RDT, and RRL. PC2 explained 34.656% of the variance (λ = 1.348) with high loadings on RAR and RGP. PC3 contributed 12.751% (λ = 0.848), primarily loading on RAR.

The PCA results were highly consistent in REP2, with three principal components (λ > 0.693) explaining 91.649% cumulative variance. PC1 dominated the analysis (69.761%, λ = 5.581) and showed strong loadings on RGR, RDT, and RSL, playing key indicators of germination performance under drought stress. PC2 (13.229%, λ = 1.058) and PC3 (8.66%, λ = 0.693) both showed significant loadings on RAR, confirming its consistent importance across replicates.

These robust and reproducible principal component analysis (PCA) predictions that RSL, RGR, RDT, and RRL are core indicators of drought stress tolerance during the germination stage of cotton need to be further confirmed.

### 2.4. Comprehensive Evaluation of Drought Tolerance and Cluster Analysis

**Figure 2:**
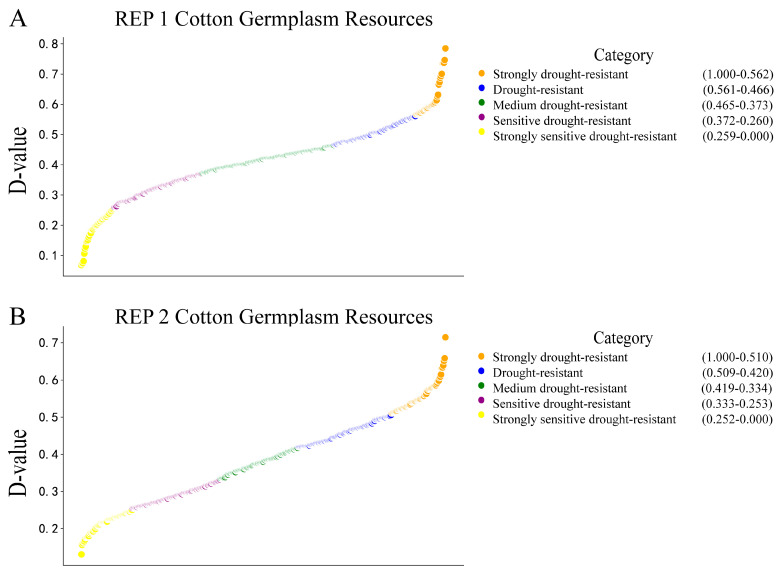
Cluster analysis of D-value from two REPs. (**A**) REP1 cotton Germplasm resources; (**B**) REP2 cotton Germplasm resources.

The D-value for REP1 ranged from 0.068 to 0.785, with an average of 0.413 and CVs being 0.279. The cotton germplasm materials were ranked based on the D-value (Table S1). The 502 cotton accessions were classified into the following categories: strongly drought-resistant with 41 varieties (8.1%); drought-resistant with 112 varieties (22.3%); medium drought-resistant with 180 varieties (35.9%); sensitive drought-resistant with 124 varieties (24.7%); and strongly sensitive drought-resistant with 45 varieties (8.9%) (Figure 2).

The D-value for REP2 ranged from 0.131 to 0.715, with an average of 0.381 and a coefficient of variation of 0.279 (Table S2); strongly drought-resistant with 76 varieties (15.1%); drought-resistant with 127 varieties (25.3%); medium drought-resistant with 107 varieties (21.3%); sensitive drought-resistant with 122 varieties (24.3%); and strongly sensitive drought-resistant with 70 varieties (13.9%).

The WDC- and CDC-values in REP1 ranged, respectively, from 0.023 to 0.881 and 0.024 to 0.872, with average values of 0.409 and 0.402 and CVs of 0.333 and 0.334. The 502 cotton accessions were ranked according to WDC- and CDC-values, and the results were the same.

The WDC- and CDC-values in REP2 ranged, respectively, from 0.158 to 0.773 and 0.168 to 0.811, with average values of 0.443 and 0.467 and CVs of 0.288 and 0.292. The results are almost the same.

There are slight differences between the D, WDC, and CDC methods, and the classification results for individual varieties may differ, but the overall results are highly consistent. The evaluation is mainly based on the D-value, with WDC and CDC as supplementary factors. Finally, HS120 was identified as a strongly drought-resistant accession, while HS491 was identified as a strongly drought-sensitive accession.

### 2.5. Gray Correlation Analysis

In REP1 (Table 8), the three evaluation methods showed highly consistent results across all eight indicators. RFW achieved the highest comprehensive evaluation score, indicating it was the most sensitive to water stress, while RGP scored the lowest. The results demonstrate that water stress treatment during germination significantly affected RFW, RSL, RRL, and RHL, showing these parameters were highly sensitive to water stress. In contrast, RAR, RGP, RGR, and RDT showed relatively weak responses to water stress.

In REP2 (Table 9), the three methods again showed a strong correlation. RSL showed the greatest water stress sensitivity and ranked highest in the comprehensive evaluation, while RHL showed the lowest sensitivity. Notably, compared with REP1, RAR and RGP exhibited significant water stress sensitivity in this stage, while RGR and RDT maintained relatively stable and insensitive responses.

In summary, RSL and RRL consistently showed significant sensitivity to water stress in both REPs and can serve as reliable indicators for early stress detection. Conversely, RGR and RDT maintained stable, less sensitive responses under stress conditions, demonstrating strong environmental adaptability.

These results provide important theoretical support for establishing a water stress evaluation system during cotton germination, with RSL and RRL serving as sensitive evaluation indicators and RGR and RDT functioning as stable reference standards.

### 2.6. Stepwise Regression Analysis

The D-value, WDC-value, and CDC-value of the two REPs were used as the dependent variables, and the DC of each index was used as the independent variable for stepwise regression analysis to establish the regression equation of drought tolerance in cotton (Table 10). The regression equations obtained by the three evaluation methods of the two REPs had coefficients of determination of R^2^ > 0.873 or more, reaching a highly significant level (*p* > 0.001). The accuracy of the D-value predictions from the equations was analyzed. The results showed that the prediction accuracy of both REPs for each variety (line) exceeded 92.52% (Supplementary File S1), indicating that the model has a good fitting degree and high prediction accuracy and can be used to evaluate the drought resistance of cotton varieties.

Further analysis revealed highly significant positive correlations among D-value, WDC-value, and CDC-value, with almost consistent results across all three evaluation methods. To efficiently screen for drought tolerance during cotton germination, indicators related to D-values, such as RFW, RHL, RAR, RGR, RGP, and RDT, should be prioritized to accelerate variety selection and breeding processes.

BLUP was employed to estimate the drought response values (D-values) of phenotypic traits across two experimental REPs (REP1 and REP2). The prediction model demonstrated high accuracy, with a mean prediction error of only 7.7% (Supplementary File S2). Based on these results, HS120 was identified as the strongly drought-accession, while HS491 was classified as the strongly drought-sensitive. These findings not only validate our previous analytical conclusions but also provide a reliable basis for subsequent investigations.

## 3. Discussion

Global water scarcity has become a major and urgent issue in agricultural development, seriously threatening the growth and development of crops, and the drought problem in China is particularly serious. Thus, the rational utilization of crops’ drought-resistant capacity is an important, effective, and economic drought-resistant measure, which has special significance in alleviating the shortage of water resources and ensuring the sustainable development of agriculture.

Seeds during the germination stage have a high demand for water, and drought stress can inhibit the water absorption capacity of seeds, thereby affecting the growth and germination of embryos [40]. PEG-6000, as a plant drought stress simulator or osmotic regulator, is often used to simulate natural soil water deficits [41]. Cotton has a certain drought resistance, and the identification of drought resistance is typically conducted during the crop germination stage. This process can be carried out indoors, making it simple to operate and relatively short in duration. This method has also been widely applied to other crops, such as tomato [42], alfalfa [43], and pea [44].

To better analyze the differences among cotton varieties under normal water supply and drought stress conditions, this study used data from two replicates (REP1 and REP2) for evaluation. Under both normal water and drought stress conditions, all measured traits exhibited significant differences with a wide range of variation, indicating that all the phenotypic traits of the 502 cotton accessions selected in this experiment were sensitive to drought in general. Crop resistance is influenced by the joint action of seed resistance heritability and the environment. Different indicators showed significant differences in sensitivity to stress, and a single indicator often failed to fully reflect crop stress resistance [45,46]. Li et al. [5] selected six phenotypic traits related to germination, such as GP, GR, FW, SL, HL, and RL, when conducting a screening for salt tolerance in the germination stage of cotton. Zhao et al. [23] selected four phenotypic traits, such as GR, GE, GI, and GDI, in conducting a drought tolerance test for soybean germination. Niu et al. [20] selected relevant physiological indicators such as fresh mass, ROS, and NPQ to evaluate the drought resistance of Hulless Barley seedlings. In the present experiment, eight phenotypic traits associated with germination were screened based on the summary of the previous studies. Badr et al. [47] conducted a simulated drought stress test for germination and seedling traits of maize and found that all the phenotypic traits of the seedlings were significantly reduced under PEG-6000 stress. The present study confirmed that all indicators showed different degrees of significant reduction under drought stress compared with the control, indicating the reliability of the data. The combination of drought stress tolerance coefficient correlation analysis, principal component analysis, affiliation function analysis, cluster analysis, gray correlation analysis, and stepwise regression analysis can reflect the drought tolerance of crops more accurately and effectively [5].

Ren et al. [35] transformed 27 physiological and biochemical indicators into five independent indices through correlation analysis and PCA and then obtained a comprehensive evaluation oft he D-value through membership function analysis. The D-values were sorted, and six drought-resistant germplasm were screened out. Li et al. [48] transformed thirteen physiological and biochemical indicators of lettuce into four independent and comprehensive indices by PCA and evaluated the drought resistance of lettuce by three evaluation methods: D-value, WDC-value, and CDC-value. There is no significant difference among the three evaluation methods [35]. This study used correlation analysis and PCA to convert eight phenotypic indicators into three independent composite indicators, retaining 89.206% (REP1) and 91.649% (REP2) of the information. The drought tolerance of 502 cotton accessions in the germination period was ranked by D-value, WDC-value, and CDC-value calculated by membership function analysis. The evaluation results of the above three evaluation methods were slightly different, but the similarity was very high. There is also a positive correlation between D-value, WDC-value, and CDC-value. Therefore, the D-value can be used as the primary evaluation indicator, while WDC-value and CDC-value can be used as subsidiary evaluation indicators for comprehensive analysis, which can better judge the drought tolerance of cotton varieties. One strongly drought-tolerant accession and one strongly drought-sensitive accession were identified.

The selection of appropriate drought resistance indicators is crucial for accurately assessing crop tolerance to water stress, as different traits exhibit varying sensitivity to drought conditions [49]. Gray correlation analysis revealed that RSL and RRL demonstrated high sensitivity to water stress, showing rapid responses, whereas RGR and RDT exhibited slower responses—a finding consistent with previous crop drought resistance studies [50]. Consequently, RSL, RRL, RGR, and RDT were prioritized as candidate indicators during preliminary screening. Stepwise regression analysis identified significant physiological and morphological correlations among RFW, RHL, RAR, RGR [23], RGP, and RDT. Specifically, RAR directly influences early germination activity, with higher RAR values facilitating embryo root emergence through the seed coat, thereby affecting both RGR and RGP. Enhanced water absorption further promotes RHL and RFW development, establishing a multidimensional response mechanism. The positive correlation and synergistic relationship between RGR, RGP, and RDT underscore the importance of rapid, efficient germination in drought resistance. Notably, RFW and RHL demonstrate a trade-off relationship where excessive RHL may compromise RFW. This multidimensional indicator system was validated through BLUP analysis, showing high prediction accuracy (92.3% concordance between observed and predicted D-values), confirming its reliability for germination-stage drought tolerance evaluation in cotton.

Drought tolerance assessment in crops constitutes a complex, multi-stage evaluation process involving diverse physiological and morphological adaptation mechanisms [51,52], and mitigation strategies vary accordingly [53]. Current cotton drought research predominantly focuses on yield-critical stages (seedling and boll development), while germination-stage tolerance remains understudied. This research gap persists despite growing evidence demonstrating the germination phase’s significant influence on subsequent crop development. The oversight likely stems from (1) the germination period’s relatively short duration and (2) greater emphasis on yield-determining growth stages. However, emerging studies underscore the germination stage’s fundamental role in establishing drought resilience that persists throughout the crop cycle.

Notably, drought tolerance exhibits developmental stage-specificity due to temporal variations in gene expression patterns [54]. Three key aspects characterize this phenomenon: (1) distinct genetic regulation between germination-stage and later-stage drought tolerance, (2) carry-over effects of germination vigor on subsequent growth performance, and (3) stage-dependent environmental stress intensity potentially confounding tolerance evaluations. These factors are critical investigations into whether germination-stage, drought-tolerant cotton germplasm maintains comparable tolerance during seedling and boll development phases.

In recent years, drought resistance screening has combined cutting-edge genomics tools with machine learning methods. GWAS have identified key genes involved in cotton germination, while single-cell RNA sequencing has revealed tissue-specific drought responses. S-G + 1D-CNN has been successfully applied to predict cotton variety and evaluate drought tolerance [55]. These advancements mark a shift toward multi-omics data integration and AI-driven predictive screening in drought tolerance assessment.

This study holds practical value for breeding applications. The established germination-stage indicators enable efficient screening, offering two main benefits: (1) reduced breeding cycle duration through early selection and (2) large-scale germplasm evaluation under controlled greenhouse conditions.

Several limitations should be noted: first, experiments were conducted only under controlled laboratory conditions, without accounting for field variables like soil characteristics and weather patterns. Second, the current indicators assess only initial 7-day germination responses, lacking evaluation of subsequent growth stages.

In conclusion, this study conducted an objective and comprehensive evaluation of 502 cotton accessions for drought tolerance during the germination period to quickly establish a set of accurate, comprehensive, and rapid identification systems of drought tolerance in cotton, provide valuable reference data for future drought tolerance screening and support the breeding and improvement of drought-tolerant cotton varieties.

## 4. Materials and Methods

### 4.1. Experimental Conditions and Plant Material

The experiment was conducted in May 2024 at Xinjiang Academy of Agricultural and Reclamation Science, using 502 cotton accessions collected by the same institution.

Uniform and full-sized cotton seeds were selected, sterilized, and soaked in 10% H_2_O_2_ for 30 min. They were then washed 3–4 times with sterile water to remove surface residue and dried with filter paper (30 cm in length and 20 cm in width), which were fully moistened with equal volumes of water solution or 15% PEG-6000 solution, respectively, and finally placed flat on the test bench. Forty seeds were placed 3 cm below the upper end of the filter paper in 2 rows (20 seeds in each row), covered with the second layer of filter paper moistened with the same concentration, and aligned to lay flat on the seeds. A moisturized zone was formed by folding the filter paper 1.5–2 cm from the top of the filter paper, which was subsequently rolled into a cylinder and secured in a germination box with a rubber band. Each box was injected 3 cm deep with the corresponding concentration, and three technical replicates were set for each treatment. The germination boxes were placed in an artificial climate chamber and maintained at a temperature of (30 + 1) °C and relative humidity of 60%, with dark incubation on the first two days and supplemental light since the third day, with the light time from 9 AM to 9 PM and the light/dark time of 12 h/12 h (L/D). On the first day, randomly select 5 seeds, weigh them, and place them in the upper right corner of the filter paper. Starting from the day after treatment, measure the weight of the seeds at the fixed position and record the number of germinated seeds on days 2, 3, 4, and 7. On the 7th day, randomly select 5 seedlings with consistent growth and measure morphological indicators such as GR, FW, SL, RL, and HL. All experiments were conducted with two independent biological REPs.

### 4.2. Measurement Indicators

Seeds water absorption rate (AR) = (mass of seed at 20 h of water absorption − mass of seed not absorbing water)/mass of seed not absorbing water × 100%.

Germination potential (GP) = number of seeds germinated on day 3/number of seeds supplied for testing × 100%.

Germination rate (GR) = number of seeds germinated on day 7/number of seeds supplied for testing × 100%.

Drought tolerance index (DT) = (GR2 + GR4 + GR7), in that GR2, GR4, and GR7 are the seed germination rates on day 2, day 4, and day 7, respectively.

Fresh weight (FW) = the seedlings were weighed on day 7 after removing them from the germination box and draining the surface water with absorbent paper.

Seeds are recognized as germinated when the radicle breaks through the seed coat and turns white.

Seedling length (SL) = root crown to the cotyledon attachment point.

Root length (RL) = root of the embryonic axis.

Hypocotyl length (HL) = seedling length − root length.

Relative drought resistance coefficient (DC) of each index = treatment index/control index × 100%.

### 4.3. Statistical Analysis

Microsoft Excel 2016 was used for the experimental data from two biological REPs. SPSS Statistics 27 was used to analyze the experimental data, including statistical description, variance analysis, correlation analysis, PCA, and stepwise regression analysis. Cluster analysis and generalized heritability visualization were performed using the R 4.4.2 packages “cluster” and “factoextra”. GraphPad Prism 10.4.1 was used for graphing, and SPSSAU. (Version 25.0) was used for gray correlation analysis.

Through PCA, eight traits were transformed into three composite indicators, and the fuzzy membership function analysis was then used to comprehensively evaluate the drought stress tolerance of 502 cotton accessions.

In the equations, Ti and CKi, respectively, represent the measured values of the ith index under drought stress and normal (control) conditions.

$$DC=\frac{Ti}{CKi}$$

$$CDC=\frac{1}{n}\sum_{i=1}^{n}DC$$

PCA analyzed DC, and the factor weight coefficient ωi of each composite indicator was calculated according to Equation. In Equation, Pi is the contribution rate of the ith composite indicator, which indicates the importance degree of the ith composite indicator among all indicators. Calculate the affiliation function value μ (xi) of each composite indicator of different varieties according to the equation, where xi, xi, max, xi, and min denote the ith composite index and the maximum and minimum values of the ith composite indicator, respectively. Based on the factor weight coefficients ωi and the value of the subordinate function μ (xi), the drought resistance comprehensive evaluation value (D-value) of each accession was calculated according to the following equations.

$$\omega i=\frac{Pi}{\sum_{i=1}^{n}Pi}$$

$$\mu (xi)=\frac{x_{i}-x_{imin}}{x_{imax}-x_{imin}}$$

$$D=\sum_{i=1}^{n}\mu (xi)\times \omega i$$

Gray correlation analysis was performed with DC values as the comparison sequence and D-values as the reference sequence to calculate the correlation degree γD between each DC and D-value. ωi (γ) and WDC were calculated for each index weight system according to the following equations, where γi is the correlation degree of each index.

$$\omega i\left(\gamma \right)=\frac{\gamma i}{\sum_{i=1}^{n}\gamma i}$$

$$WDC=\sum_{i=1}^{n}DC\times \omega i(\gamma )$$

Finally, the D-value of each cotton accession was obtained by clustering analysis using Euclidean distance and intergroup linkage method to classify the drought-resistant level.

## 5. Conclusions

This study systematically evaluated the drought tolerance of 502 cotton accessions during the germination stage by establishing a comprehensive assessment system using 15% PEG-6000 simulated drought stress with two biological REPs. The results demonstrated that drought stress significantly affected various phenotypic traits during cotton germination. Through principal component analysis, eight original indicators (including RGP and RGR) were successfully transformed into three independent principal components. Subsequent cluster analysis based on comprehensive D-values effectively classified the materials into five distinct drought tolerance categories, ranging from highly tolerant to highly sensitive.

Notably, two contrasting varieties were identified: the strongly drought-tolerant HS120 and the strongly drought-sensitive HS491, which serve as ideal materials for future mechanistic studies. Most importantly, stepwise regression analysis identified six core evaluation indicators: RFW, RHL, RAR, RGR, RGP, and RDT.

For practical applications, we recommend prioritizing RGR and RGP for early-generation selection and conducting multi-environment validation trials across at least three ecoregions. Future research should focus on (1) elucidating the molecular mechanisms underlying the observed drought tolerance variations, (2) identifying key regulatory genes, and (3) developing linked molecular markers to facilitate marker-assisted selection. These findings establish a robust framework for evaluating germination-stage drought tolerance while providing valuable genetic resources for cotton improvement programs.

## Figures and Tables

**Table 1:** Phenotypic traits of biological replicate 1 (REP 1) under normal water and drought stress.

Treatment	Coefficient	Seeds Water Absorption Rate	Germination Potential	Germination Rate	Drought Tolerance Index	Fresh Weight	Seedling Length	Hypocotyl Length	Root Length
CK	Max	0.9	1	1	0.43	0.8	20.7	8.6	14.6
	Min	0.09	0.3	0.6	0.19	0.44	9.7	2.6	6.3
	Average	0.47	0.82	0.95	0.35	0.63	16.29	6.25	10.05
	SD	0.15	0.15	0.06	0.05	0.07	1.78	0.9	1.36
	CV	32.79%	18.02%	6.04%	13.20%	10.46%	10.91%	14.38%	13.51%
DS	Max	0.55	0.78	0.83	0.35	0.44	18.8	4	14.9
	Min	−0.06	0	0	0	0	0	0	0
	Average	0.21	0.19	0.32	0.1	0.24	8.83	1.27	7.55
	SD	0.1	0.16	0.17	0.06	0.07	3.25	0.69	2.74
	CV	50.22%	85.60%	53.60%	62.27%	28.16%	36.78%	53.97%	36.35%
Comparison with the control	Variation	−0.26	−0.62	−0.62	−0.26	−0.39	−7.46	−4.98	−2.5
	Percentage variation	−55.46%	−76.49%	−65.83%	−72.73%	−61.49%	−45.80%	−79.63%	−24.87%

**Table 2:** Phenotypic traits of biological replicate 2 (REP 2) under normal water and drought stress.

Treatment	Coefficient	Seeds Water Absorption Rate	Germination Potential	Germination Rate	Drought Tolerance Index	Fresh Weight	Seedling Length	Hypocotyl Length	Root Length
CK	Max	1.066	1.000	1.000	0.42	0.948	21.1	10.1	12.8
	Min	0.065	0.317	0.617	0.16	0.278	10.4	3.9	6.1
	Average	0.609	0.816	0.931	0.343	0.63	16.2	6.9	9.2
	SD	0.16	0.13	0.066	0.047	0.069	1.3	0.9	1.1
	CV	26.26%	15.94%	7.06%	13.68%	10.98%	8.04%	13.58%	12.17%
DS	Max	0.809	0.692	0.792	0.27	0.694	18.1	6.7	14.8
	Min	0.005	0	0.042	0.01	0.098	2.7	0.2	2.4
	Average	0.286	0.214	0.328	0.093	0.295	10.6	2.2	8.5
	SD	0.107	0.128	0.141	0.045	0.097	3.7	1.5	2.5
	CV	37.31%	59.89%	42.88%	48.93%	32.70%	34.50%	67.46%	29.30%
Comparison with the control	Variation	−0.32	−0.60	−0.60	−0.25	−0.33	−5.53	−4.76	−0.77
	Percentage variation	−53.10%	−73.82%	−64.78%	−72.93%	−53.15%	−34.21%	−68.79%	−8.33%

**Table 3:** REP1 drought resistance coefficient.

Coefficient	RAR	RGP	RGR	RDT	RFW	RSL	RHL	RRL
Max	1.62	1.03	0.83	0.87	0.75	1.31	0.73	1.90
Min	−0.22	0.00	0.00	0.00	0.00	0.00	0.00	0.00
Average	0.46	0.24	0.34	0.27	0.39	0.55	0.21	0.76
SD	0.25	0.20	0.18	0.16	0.11	0.21	0.12	0.30
CV	54.03%	84.37%	52.19%	58.59%	28.68%	38.22%	56.63%	38.98%

**Table 4:** REP2 drought resistance coefficient.

Coefficient	RAR	RGP	RGR	RDT	RFW	RSL	RHL	RRL
Max	2.21	0.88	0.83	0.75	0.86	1.20	0.88	1.67
Min	0.01	0.00	0.05	0.04	0.17	0.18	0.03	0.27
Average	0.50	0.26	0.35	0.27	0.47	0.66	0.30	0.93
SD	0.23	0.15	0.14	0.12	0.14	0.22	0.19	0.31
CV	46.29%	58.47%	40.22%	43.40%	28.96%	33.76%	63.08%	32.84%

**Table 5:** Correlation analysis of DCs of REP1.

Index	RAR	RGP	RGR	RDT	RFW	RSL	RHL	RRL
RAR	1							
RGP	−0.268 **	1						
RGR	−0.117 **	0.802 **	1					
RDT	−0.192 **	0.915 **	0.929 **	1				
RFW	−0.007	0.464 **	0.595 **	0.556 **	1			
RSL	−0.027	0.509 **	0.613 **	0.576 **	0.826 **	1		
RHL	−0.040	0.453 **	0.490 **	0.493 **	0.740 **	0.769 **	1	
RRL	−0.020	0.476 **	0.600 **	0.549 **	0.757 **	0.969 **	0.617 **	1

** Indicates significant correlation at the 0.01 level.

**Table 6:** Correlation analysis of DCs of REP2.

Index	RAR	RGP	RGR	RDT	RFW	RSL	RHL	RRL
RAR	1							
RGP	−0.318 **	1						
RGR	−0.365 **	0.850 **	1					
RDT	−0.349 **	0.920 **	0.951 **	1				
RFW	−0.198 **	0.636 **	0.696 **	0.690 **	1			
RSL	−0.199 **	0.632 **	0.711 **	0.698 **	0.858 **	1		
RHL	−0.242 **	0.657 **	0.696 **	0.699 **	0.935 **	0.869 **	1	
RRL	−0.178 **	0.541 **	0.646 **	0.618 **	0.707 **	0.943 **	0.682 **	1

** Indicates significant correlation at the 0.01 level.

**Table 7:** Principal component analysis of two REPs.

Principle Factor	REP1			REP2		
	PC 1	PC 2	PC 3	PC 1	PC 2	PC 3
Eigenvalues	4.940	1.348	0.848	5.581	1.058	0.693
Contribution ratio%	41.799	34.656	12.751	69.761	13.229	8.66
Cumulative contribution ratio%	41.799	76.455	89.206	69.761	82.99	91.649
Factor weight	0.692	0.189	0.119	0.761	0.144	0.095
Eigenvector						
RAR	−0.063	0.543	0.826	−0.151	0.736	0.657
RGP	0.355	−0.429	0.199	0.359	−0.265	0.430
RGR	0.386	−0.264	0.306	0.382	−0.225	0.281
RDT	0.385	−0.362	0.276	0.383	−0.245	0.361
RFW	0.378	0.282	−0.125	0.376	0.242	−0.149
RSL	0.404	0.303	−0.173	0.388	0.300	−0.233
RHL	0.347	0.264	−0.210	0.378	0.198	−0.169
RRL	0.382	0.282	−0.138	0.348	0.300	−0.262

**Table 8:** REP1 gray correlation analysis based on D, WDC, and CDC.

Index	D Correlation Degree	Rank	WDC Correlation Degree	Rank	CDC Correlation Degree	Rank
RAR	0.808	7	0.74	5	0.741	5
RGP	0.758	8	0.72	8	0.721	8
RGR	0.843	5	0.738	6	0.738	6
RDT	0.828	6	0.735	7	0.736	7
RFW	0.944	1	0.764	1	0.764	1
RSL	0.939	2	0.756	2	0.756	2
RHL	0.868	4	0.742	4	0.742	4
RRL	0.923	3	0.754	3	0.754	3

**Table 9:** REP2 gray correlation analysis based on D, WDC, and CDC.

Index	D Correlation Degree	Rank	WDC Correlation Degree	Rank	CDC Correlation Degree	Rank
RAR	0.826	1	0.820	3	0.817	3
RGP	0.821	4	0.818	4	0.816	4
RGR	0.820	6	0.817	6	0.815	5
RDT	0.822	3	0.817	5	0.814	6
RFW	0.812	7	0.808	7	0.806	7
RSL	0.821	5	0.821	1	0.819	1
RHL	0.806	8	0.802	8	0.801	8
RRL	0.822	2	0.820	2	0.819	2

**Table 10:** Stepwise regression equation.

REP	Dependent Variable	Multiple Stepwise Regression Equation	r	R^2^	Correlation Coefficient		
					D-Value	WDC-Value	CDC-Value
REP1	D-value	Y = 0.056 + 0.587 × RFW + 0.37 × RHL + 0.067 × RAR + 0.064 × RGR	0.949	0.901	1	0.930 **	0.918 **
	WDC-value	Y = 0.002 + 0.458 × RFW + 0.255 × RGR + 0.12 × RAR + 0.241 × RHL + 0.156 × RGP	0.950	0.903		1	0.999 **
	CDC-value	Y = 0.002 + 0.255 × RGR + 0.425 × RDT + 0.128 × RAR + 0.173 × RGP + 0.232 × RHL	0.954	0.911			1
REP2	D-value	Y = 0.62 × RFW − 0.027 + 0.25 × RGP + 0.108 × RAR	0.952	0.906	1	0.996 **	0.996 **
	WDC-value	Y = 0.74 × RDT − 0.018 + 0.332 × RGP + 0.133 × RAR	0.934	0.873		1	0.999 **
	CDC-value	Y = 0.013 + 0.663 × RDT + 0.351 × RGP + 0.107 × RAR	0.938	0.879			1

** Indicates significant correlation at the 0.01 level.

## Data Availability

Data are contained within the article and Supplementary Materials.

## Supplementary Materials

The following supporting information can be downloaded at: https://www.mdpi.com/article/10.3390/plants14142191/s1, Table S1: D-value, WDC-value and CDC-value of REP 1 in 502 germplasm resources; Table S2: D-value, WDC-value and CDC-value of REP 2 in 502 germplasm resources; Table S3: BLUP Predicting D values with actual error;.
