# Supplementary material for: Screening and Comprehensive Evaluation of Drought Resistance in Cotton Germplasm Resources at the Germination Stage
Source: Plants (Basel). 2025 Jul 15;14(14):2191. doi: 10.3390/plants14142191 (PMC12299762; doi:10.3390/plants14142191)
Supplement: Supplementary file 1 [file plants-14-02191-s001.zip › plants-3738055-supplementary/plants-3738055-supplementary/Supplement/Supplementary File S1.pdf]

Table S1. D-value, WDC-value and CDC-value of REP 1 in 502 germplasm resources

Code	REP 1											
	Subordinate function value			WDC-value	Rank	CDC-value	Rank	D-value	Rank	Predicted D-value	Absolute value of error	forecast accuracy
	μ1	μ2	μ3									
HS1	0.575	0.304	40.42%	0.494	139	0.481	148	0.503	102	0.457	0.047	89.81%
HS2	0.617	0.247	44.52%	0.497	134	0.483	143	0.527	77	0.477	0.050	89.53%
HS3	0.377	0.401	81.21%	0.478	160	0.477	153	0.433	219	0.430	0.004	99.15%
HS4	0.443	0.160	28.85%	0.317	380	0.304	392	0.371	338	0.311	0.060	80.55%
HS5	0.481	0.170	17.27%	0.299	398	0.288	403	0.385	316	0.410	0.025	93.95%
HS6	0.297	0.151	31.03%	0.201	466	0.194	470	0.271	452	0.289	0.018	93.63%
HS7	0.229	0.212	40.67%	0.210	463	0.206	462	0.247	461	0.241	0.006	97.41%
HS8	0.242	0.192	25.32%	0.188	473	0.182	474	0.234	466	0.204	0.030	85.41%
HS9	0.521	0.613	37.48%	0.560	65	0.556	59	0.521	82	0.603	0.082	86.37%
HS10	0.568	0.381	80.25%	0.586	43	0.578	42	0.561	44	0.548	0.012	97.76%
HS11	0.435	0.263	82.50%	0.431	226	0.430	215	0.449	184	0.493	0.044	91.01%
HS12	0.149	0.224	46.70%	0.170	480	0.171	480	0.201	481	0.209	0.008	96.07%
HS13	0.402	0.159	47.85%	0.322	377	0.313	381	0.365	346	0.319	0.046	85.53%
HS14	0.533	0.329	36.65%	0.451	200	0.440	205	0.475	142	0.490	0.015	97.00%
HS15	0.273	0.265	34.33%	0.255	435	0.250	434	0.280	441	0.267	0.013	95.30%
HS16	0.049	0.238	57.84%	0.129	489	0.134	486	0.148	494	0.173	0.025	85.40%
HS17	0.225	0.291	41.62%	0.231	450	0.229	451	0.260	457	0.303	0.043	85.82%
HS18	0.375	0.265	47.99%	0.349	343	0.341	341	0.366	345	0.343	0.023	93.29%
HS19	0.535	0.632	25.84%	0.584	46	0.578	43	0.520	83	0.543	0.023	95.81%
HS20	0.653	0.260	9.17%	0.466	171	0.446	195	0.512	89	0.465	0.047	89.89%
HS21	0.449	0.160	26.46%	0.328	371	0.315	376	0.372	336	0.292	0.081	72.36%
HS22	0.563	0.611	16.59%	0.576	48	0.567	49	0.525	79	0.523	0.002	99.69%
HS23	0.464	0.371	31.66%	0.418	244	0.408	247	0.429	229	0.433	0.004	99.08%

HS24	0.290	0.553	40.50%	0.401	271	0.399	263	0.353	363	0.405	0.051	87.30%
HS25	0.302	0.639	24.59%	0.442	212	0.438	207	0.359	356	0.348	0.011	96.91%
HS26	0.372	0.375	81.36%	0.447	205	0.448	189	0.425	237	0.470	0.045	90.34%
HS27	0.241	0.507	16.71%	0.306	394	0.307	388	0.283	438	0.352	0.069	80.28%
HS28	0.519	0.367	42.44%	0.472	165	0.464	165	0.479	135	0.490	0.011	97.72%
HS29	0.401	0.775	19.17%	0.574	49	0.570	46	0.447	190	0.415	0.031	92.50%
HS30	0.452	0.674	20.02%	0.539	88	0.535	80	0.464	158	0.487	0.023	95.23%
HS31	0.148	1.000	25.28%	0.510	117	0.517	100	0.321	403	0.364	0.042	88.44%
HS32	0.276	0.816	16.96%	0.504	126	0.503	116	0.365	347	0.355	0.010	97.14%
HS33	0.487	0.731	27.05%	0.630	19	0.623	18	0.507	98	0.431	0.076	82.40%
HS34	0.653	0.416	25.50%	0.540	83	0.530	86	0.561	43	0.607	0.046	92.41%
HS35	0.666	0.449	25.83%	0.571	51	0.560	55	0.577	34	0.597	0.021	96.53%
HS36	0.516	0.343	22.55%	0.419	242	0.409	244	0.449	183	0.446	0.003	99.39%
HS37	0.587	0.448	87.95%	0.633	17	0.629	17	0.595	22	0.615	0.020	96.81%
HS38	0.780	0.201	27.66%	0.518	106	0.501	122	0.611	14	0.639	0.028	95.62%
HS39	0.607	0.231	37.46%	0.455	196	0.442	202	0.509	94	0.490	0.018	96.24%
HS40	0.525	0.276	34.45%	0.399	273	0.387	281	0.457	167	0.509	0.053	89.66%
HS41	0.416	0.448	25.72%	0.403	266	0.397	265	0.403	277	0.465	0.062	86.59%
HS42	0.380	0.343	29.42%	0.352	337	0.345	338	0.363	351	0.345	0.018	94.76%
HS43	0.328	0.243	41.88%	0.283	416	0.278	414	0.323	402	0.345	0.022	93.58%
HS44	0.620	0.586	36.26%	0.628	21	0.618	21	0.583	30	0.603	0.021	96.59%
HS45	0.445	0.633	29.87%	0.544	78	0.539	77	0.463	160	0.452	0.012	97.44%
HS46	0.372	0.395	73.56%	0.446	206	0.446	196	0.420	251	0.441	0.021	95.16%
HS47	0.658	0.318	46.21%	0.551	75	0.540	74	0.570	39	0.568	0.002	99.68%
HS48	0.478	0.175	41.83%	0.350	339	0.339	345	0.414	261	0.422	0.009	97.96%
HS49	0.520	0.138	38.15%	0.386	295	0.371	305	0.432	222	0.340	0.091	73.16%
HS50	0.555	0.223	30.27%	0.415	247	0.402	260	0.462	162	0.433	0.029	93.28%
HS51	0.403	0.122	40.63%	0.267	425	0.260	428	0.350	368	0.378	0.028	92.49%

HS52	0.266	0.975	23.28%	0.566	57	0.568	48	0.396	296	0.435	0.039	91.07%
HS53	0.507	0.405	39.41%	0.490	144	0.479	151	0.475	144	0.445	0.029	93.40%
HS54	0.429	0.415	100.00%	0.540	82	0.542	72	0.494	112	0.515	0.021	95.86%
HS55	0.243	0.373	35.41%	0.277	418	0.273	418	0.281	439	0.303	0.022	92.69%
HS56	0.347	0.393	22.05%	0.338	361	0.332	358	0.340	378	0.348	0.007	97.90%
HS57	0.664	0.342	17.25%	0.528	98	0.512	106	0.545	60	0.497	0.048	90.31%
HS58	0.371	0.380	29.87%	0.366	320	0.360	316	0.364	349	0.360	0.004	99.00%
HS59	0.244	0.789	25.22%	0.460	185	0.462	169	0.348	372	0.391	0.044	88.86%
HS60	0.562	0.484	24.96%	0.526	100	0.515	101	0.510	91	0.511	0.001	99.83%
HS61	0.449	0.232	31.25%	0.346	351	0.335	353	0.392	304	0.375	0.017	95.37%
HS62	0.246	0.166	38.24%	0.188	472	0.184	473	0.247	462	0.265	0.018	93.28%
HS63	0.153	0.262	37.45%	0.179	478	0.177	478	0.200	482	0.203	0.003	98.41%
HS64	0.124	0.196	30.79%	0.115	494	0.114	494	0.159	492	0.157	0.002	98.61%
HS65	0.465	0.333	37.82%	0.434	220	0.424	223	0.430	227	0.380	0.050	86.82%
HS66	0.452	0.304	40.62%	0.397	279	0.391	275	0.419	253	0.426	0.007	98.34%
HS67	0.403	0.434	53.91%	0.449	202	0.445	198	0.425	236	0.457	0.032	93.06%
HS68	0.317	0.196	20.54%	0.226	453	0.218	457	0.281	440	0.268	0.013	95.28%
HS69	0.465	0.275	43.42%	0.385	297	0.377	298	0.425	235	0.458	0.033	92.83%
HS70	0.271	0.444	38.11%	0.338	363	0.335	351	0.316	409	0.326	0.010	96.97%
HS71	0.389	0.413	48.01%	0.415	248	0.408	246	0.404	274	0.414	0.010	97.63%
HS72	0.444	0.417	30.35%	0.423	233	0.415	234	0.422	250	0.438	0.016	96.45%
HS73	0.500	0.619	45.78%	0.590	41	0.590	35	0.517	84	0.553	0.036	93.53%
HS74	0.355	0.332	46.64%	0.352	336	0.347	334	0.364	350	0.388	0.024	93.74%
HS75	0.523	0.098	35.37%	0.345	352	0.332	359	0.423	246	0.387	0.036	90.69%
HS76	0.311	0.247	24.27%	0.240	444	0.233	446	0.291	429	0.325	0.034	89.57%
HS77	0.382	0.373	28.41%	0.361	324	0.354	324	0.368	343	0.385	0.017	95.71%
HS78	0.447	0.242	34.40%	0.342	355	0.332	357	0.396	294	0.420	0.023	94.41%
HS79	0.582	0.202	38.30%	0.418	243	0.405	252	0.486	124	0.502	0.016	96.77%

HS80	0.478	0.299	20.79%	0.376	312	0.364	313	0.412	264	0.406	0.006	98.53%
HS81	0.404	0.503	56.00%	0.487	150	0.485	139	0.441	202	0.468	0.026	94.35%
HS82	0.545	0.265	51.70%	0.455	197	0.445	197	0.489	120	0.498	0.009	98.19%
HS83	0.516	0.351	48.37%	0.471	167	0.461	170	0.481	131	0.489	0.008	98.41%
HS84	0.538	0.302	45.25%	0.469	168	0.456	175	0.483	127	0.441	0.042	90.38%
HS85	0.381	0.419	43.49%	0.411	258	0.404	255	0.394	300	0.390	0.004	99.01%
HS86	0.539	0.139	40.24%	0.379	306	0.366	311	0.447	189	0.427	0.020	95.37%
HS87	0.276	0.236	49.40%	0.268	424	0.264	424	0.294	427	0.297	0.003	99.11%
HS88	0.407	0.372	64.21%	0.441	213	0.435	210	0.429	231	0.445	0.016	96.34%
HS89	0.549	0.159	30.14%	0.373	313	0.358	319	0.446	192	0.437	0.009	97.84%
HS90	0.399	0.699	47.48%	0.570	53	0.567	50	0.464	156	0.479	0.015	96.96%
HS91	0.840	0.415	34.72%	0.683	8	0.668	9	0.701	6	0.724	0.023	96.87%
HS92	0.468	0.092	25.97%	0.254	437	0.242	438	0.372	335	0.467	0.094	79.78%
HS93	0.633	0.389	62.60%	0.602	33	0.593	33	0.586	29	0.571	0.015	97.33%
HS94	0.574	0.718	39.12%	0.683	9	0.676	8	0.580	32	0.574	0.005	99.05%
HS95	0.447	0.349	41.13%	0.403	265	0.395	267	0.424	239	0.464	0.040	91.41%
HS96	0.156	0.187	36.04%	0.131	488	0.130	489	0.186	487	0.220	0.034	84.59%
HS97	0.526	0.158	45.82%	0.378	307	0.367	309	0.449	185	0.468	0.019	95.92%
HS98	0.571	0.605	30.91%	0.591	40	0.583	39	0.546	59	0.588	0.042	92.84%
HS99	0.482	0.372	52.83%	0.464	176	0.457	174	0.467	153	0.490	0.023	95.33%
HS100	0.530	0.617	48.79%	0.607	30	0.600	27	0.541	63	0.573	0.032	94.49%
HS101	0.426	0.232	48.23%	0.355	331	0.349	331	0.396	295	0.403	0.007	98.30%
HS102	0.602	0.485	18.06%	0.540	84	0.529	88	0.530	72	0.547	0.017	96.94%
HS103	0.503	0.876	24.20%	0.695	7	0.689	7	0.543	62	0.514	0.029	94.38%
HS104	0.185	0.259	34.68%	0.194	471	0.191	471	0.218	474	0.207	0.011	94.55%
HS105	0.196	0.282	30.65%	0.199	468	0.197	467	0.226	471	0.244	0.019	92.29%
HS106	0.355	0.575	19.53%	0.434	222	0.429	217	0.377	327	0.374	0.003	99.12%
HS107	0.003	0.210	27.79%	0.036	500	0.038	500	0.075	500	0.077	0.002	96.89%

HS108	0.576	0.220	35.28%	0.423	235	0.409	245	0.482	129	0.489	0.007	98.63%
HS109	0.213	0.367	38.51%	0.260	432	0.258	431	0.263	455	0.289	0.027	90.83%
HS110	0.398	0.138	0.00%	0.211	462	0.198	466	0.302	423	0.289	0.012	95.72%
HS111	0.785	0.148	35.62%	0.551	74	0.529	87	0.613	13	0.526	0.088	83.32%
HS112	0.458	0.357	45.22%	0.446	207	0.438	208	0.438	210	0.411	0.027	93.41%
HS113	0.554	0.202	47.11%	0.421	239	0.410	242	0.478	140	0.486	0.008	98.30%
HS114	0.623	0.524	35.95%	0.609	28	0.599	29	0.573	35	0.580	0.006	98.89%
HS115	0.668	0.456	34.20%	0.602	34	0.588	36	0.589	25	0.580	0.009	98.53%
HS116	0.517	0.255	28.07%	0.395	282	0.381	292	0.439	206	0.431	0.008	98.12%
HS117	0.626	0.600	33.62%	0.629	20	0.621	19	0.587	28	0.640	0.053	91.67%
HS118	0.617	0.450	18.21%	0.551	72	0.536	78	0.534	70	0.493	0.040	91.82%
HS119	0.445	0.310	39.16%	0.398	277	0.390	277	0.413	262	0.400	0.013	96.80%
HS120	0.733	0.226	42.21%	0.559	67	0.541	73	0.601	18	0.550	0.050	90.91%
HS121	0.263	0.285	34.19%	0.243	440	0.240	439	0.277	449	0.312	0.036	88.53%
HS122	0.331	0.282	25.34%	0.275	420	0.267	422	0.312	412	0.333	0.020	93.85%
HS123	0.322	0.532	33.41%	0.426	232	0.423	227	0.363	353	0.340	0.023	93.16%
HS124	0.305	0.424	17.24%	0.315	385	0.310	384	0.311	414	0.331	0.019	94.13%
HS125	0.360	0.711	44.64%	0.549	77	0.550	65	0.437	214	0.455	0.019	95.88%
HS126	0.155	0.247	49.88%	0.180	477	0.181	475	0.213	476	0.258	0.045	82.69%
HS127	0.681	0.000	16.02%	0.345	353	0.331	363	0.490	117	0.489	0.001	99.82%
HS128	0.266	0.411	36.93%	0.316	381	0.314	377	0.306	417	0.331	0.026	92.28%
HS129	0.253	0.350	35.71%	0.263	428	0.260	426	0.284	436	0.345	0.062	82.13%
HS130	0.303	0.320	34.00%	0.302	397	0.296	395	0.310	415	0.297	0.013	95.66%
HS131	0.435	0.267	33.22%	0.358	328	0.348	332	0.391	307	0.381	0.010	97.32%
HS132	0.332	0.354	36.17%	0.338	360	0.333	354	0.340	379	0.331	0.009	97.18%
HS133	0.229	0.319	32.08%	0.228	452	0.224	452	0.257	458	0.309	0.052	83.23%
HS134	0.476	0.540	28.08%	0.512	115	0.504	114	0.465	155	0.456	0.009	98.12%
HS135	0.251	0.402	34.61%	0.296	401	0.293	397	0.291	430	0.325	0.034	89.53%

HS136	0.471	0.340	27.30%	0.413	255	0.402	259	0.423	247	0.392	0.031	92.16%
HS137	0.545	0.564	20.74%	0.555	70	0.544	70	0.508	95	0.497	0.011	97.77%
HS138	0.585	0.378	33.31%	0.540	85	0.525	91	0.516	86	0.424	0.091	78.47%
HS139	0.546	0.264	13.12%	0.402	268	0.387	280	0.443	200	0.404	0.039	90.35%
HS140	0.321	0.492	24.39%	0.359	326	0.354	321	0.344	376	0.397	0.053	86.70%
HS141	0.451	0.325	18.95%	0.369	317	0.359	318	0.396	297	0.394	0.002	99.58%
HS142	0.404	0.182	11.48%	0.243	443	0.234	445	0.328	396	0.358	0.030	91.52%
HS143	0.460	0.160	29.73%	0.305	395	0.294	396	0.384	319	0.399	0.015	96.32%
HS144	0.003	0.209	26.45%	0.033	501	0.035	501	0.073	501	0.075	0.002	97.05%
HS145	0.423	0.139	30.00%	0.272	423	0.262	425	0.355	359	0.376	0.021	94.33%
HS146	0.343	0.167	5.06%	0.184	475	0.174	479	0.275	450	0.329	0.054	83.55%
HS147	0.326	0.389	30.46%	0.335	367	0.331	362	0.335	385	0.363	0.027	92.52%
HS148	0.269	0.389	27.24%	0.294	403	0.291	401	0.292	428	0.300	0.008	97.45%
HS149	0.374	0.304	31.21%	0.336	364	0.328	366	0.354	362	0.330	0.024	92.86%
HS150	0.451	0.237	38.38%	0.367	319	0.357	320	0.403	279	0.383	0.019	95.01%
HS151	0.424	0.356	59.01%	0.423	234	0.417	233	0.431	223	0.465	0.034	92.61%
HS152	0.438	0.494	84.12%	0.565	58	0.564	52	0.497	109	0.489	0.008	98.39%
HS153	0.357	0.406	51.58%	0.391	285	0.389	278	0.385	315	0.447	0.062	86.23%
HS154	0.402	0.241	41.50%	0.339	359	0.329	365	0.373	333	0.356	0.017	95.20%
HS155	0.203	0.252	27.12%	0.182	476	0.178	476	0.220	473	0.227	0.007	97.03%
HS156	0.263	0.172	19.04%	0.160	482	0.153	482	0.238	465	0.287	0.049	82.86%
HS157	0.337	0.308	28.91%	0.291	406	0.284	406	0.326	398	0.364	0.038	89.51%
HS158	0.451	0.196	67.13%	0.394	283	0.387	284	0.429	230	0.426	0.002	99.45%
HS159	0.489	0.159	34.42%	0.327	372	0.317	373	0.409	268	0.457	0.048	89.48%
HS160	0.600	0.065	28.67%	0.350	340	0.335	352	0.461	165	0.471	0.009	98.00%
HS161	0.400	0.432	41.96%	0.414	250	0.407	248	0.408	271	0.450	0.042	90.70%
HS162	0.306	0.561	37.09%	0.405	263	0.404	253	0.362	354	0.436	0.074	83.06%
HS163	0.563	0.892	32.80%	0.722	6	0.720	6	0.598	20	0.678	0.080	88.19%

HS164	0.791	0.561	16.90%	0.670	11	0.659	11	0.673	9	0.763	0.090	88.20%
HS165	0.487	0.381	41.55%	0.458	188	0.452	184	0.459	166	0.473	0.014	96.97%
HS166	0.563	0.495	56.82%	0.594	39	0.585	38	0.551	54	0.550	0.001	99.80%
HS167	0.608	0.431	39.46%	0.568	55	0.556	60	0.549	56	0.509	0.041	92.01%
HS168	0.534	0.303	38.36%	0.455	198	0.442	204	0.473	147	0.433	0.040	90.86%
HS169	0.233	0.466	28.48%	0.310	389	0.307	390	0.283	437	0.302	0.019	93.66%
HS170	0.563	0.132	25.19%	0.344	354	0.331	361	0.444	195	0.485	0.041	91.57%
HS171	0.720	0.293	28.10%	0.536	91	0.520	96	0.587	26	0.595	0.008	98.69%
HS172	0.184	0.298	34.62%	0.208	464	0.205	463	0.225	472	0.225	0.000	99.96%
HS173	0.403	0.393	62.75%	0.457	191	0.452	185	0.428	232	0.411	0.017	95.87%
HS174	0.350	0.169	36.25%	0.256	434	0.248	437	0.317	408	0.328	0.011	96.75%
HS175	0.325	0.159	52.50%	0.265	427	0.260	429	0.318	407	0.329	0.011	96.56%
HS176	0.446	0.291	30.81%	0.360	325	0.351	327	0.400	288	0.428	0.028	93.46%
HS177	0.403	0.312	27.24%	0.347	346	0.337	349	0.370	340	0.355	0.015	95.76%
HS178	0.276	0.786	51.86%	0.529	97	0.532	84	0.401	284	0.458	0.057	87.59%
HS179	0.529	0.452	33.83%	0.517	108	0.506	112	0.491	115	0.461	0.031	93.34%
HS180	0.575	0.541	18.86%	0.570	54	0.557	58	0.522	81	0.481	0.042	91.29%
HS181	0.307	0.720	28.65%	0.506	122	0.502	117	0.383	322	0.322	0.060	81.24%
HS182	0.250	0.604	35.21%	0.405	264	0.403	258	0.329	394	0.329	0.001	99.79%
HS183	0.397	0.502	27.08%	0.444	210	0.437	209	0.402	281	0.379	0.023	93.83%
HS184	0.156	0.494	22.51%	0.267	426	0.266	423	0.228	469	0.236	0.008	96.56%
HS185	0.005	0.202	21.75%	0.023	502	0.024	502	0.068	502	0.069	0.002	97.62%
HS186	0.256	0.306	15.18%	0.217	458	0.212	458	0.253	459	0.287	0.034	88.28%
HS187	0.183	0.201	22.72%	0.136	486	0.133	487	0.192	485	0.203	0.011	94.39%
HS188	0.223	0.156	21.33%	0.128	490	0.124	492	0.209	478	0.252	0.043	82.86%
HS189	0.167	0.242	10.35%	0.117	493	0.114	493	0.174	489	0.218	0.045	79.58%
HS190	0.000	0.218	33.35%	0.048	499	0.051	499	0.081	499	0.084	0.003	96.34%
HS191	0.492	0.467	42.17%	0.486	153	0.479	150	0.479	134	0.532	0.054	89.94%

HS192	0.496	0.593	19.24%	0.520	104	0.515	102	0.478	139	0.514	0.036	93.03%
HS193	0.393	0.404	33.47%	0.389	288	0.384	286	0.388	313	0.411	0.023	94.38%
HS194	0.551	0.151	36.55%	0.387	294	0.373	301	0.454	173	0.421	0.033	92.28%
HS195	0.580	0.283	44.28%	0.484	156	0.470	160	0.508	97	0.472	0.036	92.45%
HS196	0.614	0.493	36.23%	0.585	44	0.574	44	0.561	42	0.562	0.001	99.87%
HS197	0.466	0.153	35.76%	0.322	378	0.312	382	0.394	301	0.403	0.009	97.71%
HS198	0.316	0.620	43.82%	0.476	162	0.475	155	0.388	314	0.395	0.007	98.27%
HS199	0.050	0.216	39.07%	0.083	497	0.086	497	0.122	497	0.152	0.030	80.14%
HS200	0.479	0.505	21.84%	0.475	164	0.469	161	0.453	175	0.480	0.027	94.46%
HS201	0.689	0.124	23.20%	0.420	240	0.406	250	0.528	74	0.557	0.030	94.64%
HS202	0.467	0.133	39.53%	0.334	368	0.325	368	0.395	298	0.352	0.043	87.66%
HS203	0.555	0.275	39.86%	0.456	194	0.442	201	0.484	126	0.443	0.041	90.81%
HS204	0.421	0.211	47.39%	0.347	349	0.337	348	0.388	312	0.376	0.012	96.93%
HS205	0.430	0.271	56.84%	0.388	291	0.382	289	0.416	257	0.431	0.014	96.67%
HS206	0.371	0.187	39.25%	0.287	409	0.280	411	0.339	380	0.334	0.005	98.52%
HS207	0.633	0.252	34.80%	0.485	154	0.470	159	0.527	76	0.499	0.028	94.36%
HS208	0.377	0.258	43.84%	0.323	375	0.316	375	0.362	355	0.388	0.026	93.28%
HS209	0.456	0.462	32.18%	0.461	182	0.454	181	0.441	204	0.442	0.001	99.83%
HS210	0.663	0.097	46.13%	0.450	201	0.435	212	0.532	71	0.509	0.023	95.56%
HS211	0.775	0.810	39.46%	0.881	1	0.872	1	0.736	4	0.677	0.059	91.23%
HS212	0.276	0.241	43.71%	0.257	433	0.251	433	0.289	432	0.298	0.009	97.11%
HS213	0.900	0.129	34.21%	0.590	42	0.569	47	0.688	8	0.672	0.016	97.57%
HS214	0.706	0.338	15.48%	0.516	110	0.503	115	0.571	38	0.609	0.038	93.79%
HS215	0.498	0.473	41.05%	0.487	149	0.484	141	0.483	128	0.543	0.061	88.80%
HS216	0.424	0.545	39.21%	0.487	152	0.482	145	0.443	199	0.474	0.031	93.43%
HS217	0.132	0.295	55.07%	0.208	465	0.210	459	0.213	477	0.234	0.022	90.75%
HS218	0.421	0.305	68.39%	0.417	245	0.413	237	0.431	224	0.466	0.035	92.47%
HS219	0.295	0.947	8.05%	0.533	92	0.533	83	0.393	302	0.467	0.074	84.17%

HS220	0.349	0.231	39.25%	0.294	404	0.288	404	0.332	392	0.323	0.009	97.33%
HS221	0.524	0.407	23.60%	0.435	218	0.423	224	0.468	152	0.543	0.075	86.20%
HS222	0.412	0.624	43.28%	0.520	105	0.520	97	0.455	169	0.491	0.036	92.68%
HS223	0.412	0.319	47.96%	0.377	308	0.371	306	0.402	280	0.438	0.035	91.93%
HS224	0.333	0.389	34.29%	0.346	350	0.340	343	0.345	375	0.358	0.013	96.50%
HS225	0.337	0.270	56.66%	0.316	382	0.313	378	0.352	366	0.404	0.052	87.04%
HS226	0.382	0.298	43.16%	0.347	347	0.341	342	0.372	334	0.390	0.018	95.40%
HS227	0.296	0.207	47.99%	0.251	438	0.249	435	0.301	424	0.335	0.033	90.00%
HS228	0.290	0.361	50.52%	0.323	374	0.322	369	0.329	393	0.378	0.049	87.10%
HS229	0.212	0.310	38.89%	0.229	451	0.229	450	0.252	460	0.289	0.038	86.99%
HS230	0.147	0.322	58.79%	0.233	448	0.236	442	0.232	467	0.256	0.023	90.90%
HS231	0.278	0.260	50.93%	0.287	412	0.284	407	0.302	422	0.291	0.011	96.11%
HS232	0.165	0.221	43.33%	0.170	481	0.170	481	0.207	479	0.224	0.016	92.72%
HS233	0.172	0.281	60.72%	0.232	449	0.233	447	0.244	463	0.275	0.031	88.72%
HS234	0.294	0.256	30.36%	0.240	445	0.236	443	0.288	434	0.329	0.041	87.57%
HS235	0.404	0.650	23.56%	0.509	119	0.510	109	0.431	225	0.458	0.028	93.94%
HS236	0.257	0.454	52.82%	0.355	332	0.354	322	0.327	397	0.358	0.032	91.09%
HS237	0.114	0.232	42.98%	0.149	484	0.150	483	0.174	490	0.171	0.003	98.23%
HS238	0.228	0.431	18.47%	0.283	415	0.280	410	0.261	456	0.252	0.009	96.27%
HS239	0.543	0.287	10.21%	0.406	262	0.393	272	0.442	201	0.402	0.041	89.85%
HS240	0.385	0.502	37.98%	0.459	187	0.457	173	0.407	272	0.384	0.023	94.08%
HS241	0.609	0.278	18.60%	0.463	178	0.450	187	0.496	110	0.434	0.062	85.63%
HS242	0.508	0.317	45.06%	0.456	195	0.444	200	0.465	154	0.422	0.043	89.70%
HS243	0.820	0.048	8.64%	0.456	193	0.434	213	0.587	27	0.557	0.030	94.69%
HS244	0.418	0.215	52.09%	0.353	333	0.345	337	0.392	305	0.388	0.004	99.08%
HS245	0.409	0.337	48.17%	0.398	276	0.390	276	0.404	276	0.398	0.006	98.51%
HS246	0.328	0.235	31.84%	0.262	430	0.256	432	0.309	416	0.322	0.013	95.97%
HS247	0.566	0.123	33.26%	0.389	290	0.372	302	0.454	171	0.391	0.063	83.97%

HS248	0.333	0.264	69.36%	0.347	345	0.346	335	0.363	352	0.386	0.023	94.15%
HS249	0.586	0.381	31.14%	0.523	102	0.509	110	0.515	88	0.455	0.060	86.84%
HS250	0.387	0.347	30.65%	0.358	327	0.350	328	0.370	341	0.368	0.002	99.43%
HS251	0.550	0.278	32.50%	0.438	214	0.426	222	0.472	148	0.447	0.025	94.45%
HS252	0.492	0.465	42.28%	0.506	124	0.500	123	0.478	137	0.470	0.008	98.20%
HS253	0.537	0.303	63.84%	0.492	141	0.483	144	0.505	101	0.501	0.004	99.21%
HS254	0.606	0.312	39.45%	0.516	109	0.502	119	0.526	78	0.475	0.050	89.38%
HS255	0.373	0.331	71.19%	0.414	251	0.411	240	0.406	273	0.409	0.003	99.21%
HS256	0.489	0.230	55.85%	0.412	257	0.404	256	0.448	186	0.442	0.006	98.64%
HS257	0.568	0.486	55.05%	0.605	31	0.598	30	0.551	55	0.512	0.039	92.36%
HS258	0.119	0.271	53.98%	0.184	474	0.187	472	0.198	483	0.215	0.018	91.87%
HS259	0.467	0.112	54.50%	0.319	379	0.317	374	0.409	270	0.444	0.035	92.20%
HS260	0.419	0.377	58.26%	0.436	216	0.429	216	0.431	226	0.445	0.015	96.72%
HS261	0.044	0.218	28.72%	0.060	498	0.062	498	0.106	498	0.142	0.036	74.82%
HS262	0.075	0.251	24.80%	0.093	496	0.094	496	0.129	496	0.148	0.020	86.77%
HS263	0.346	0.328	40.54%	0.339	358	0.332	355	0.349	370	0.348	0.001	99.69%
HS264	0.457	0.391	39.50%	0.453	199	0.444	199	0.437	211	0.392	0.046	88.35%
HS265	0.492	0.186	47.96%	0.362	323	0.354	323	0.432	220	0.484	0.052	89.28%
HS266	0.561	0.109	27.07%	0.335	366	0.322	370	0.441	203	0.480	0.038	92.00%
HS267	0.290	0.192	34.68%	0.213	461	0.208	461	0.279	446	0.336	0.058	82.86%
HS268	0.379	0.223	40.48%	0.299	399	0.292	400	0.353	365	0.388	0.036	90.79%
HS269	0.476	0.276	45.78%	0.413	253	0.404	254	0.436	216	0.420	0.017	96.06%
HS270	0.434	0.250	58.82%	0.401	270	0.394	270	0.417	254	0.392	0.026	93.46%
HS271	0.233	0.280	53.83%	0.261	431	0.260	427	0.278	447	0.295	0.017	94.13%
HS272	0.598	0.446	48.69%	0.556	68	0.543	71	0.556	48	0.597	0.041	93.10%
HS273	0.530	0.449	55.45%	0.549	76	0.539	76	0.517	85	0.501	0.016	96.79%
HS274	0.175	0.184	25.40%	0.136	487	0.132	488	0.186	486	0.168	0.019	88.85%
HS275	0.804	0.132	37.96%	0.572	50	0.550	64	0.626	12	0.522	0.104	80.05%

HS276	0.484	0.305	32.75%	0.399	274	0.387	282	0.432	221	0.441	0.010	97.81%
HS277	0.354	0.645	47.91%	0.506	123	0.504	113	0.424	240	0.446	0.022	94.98%
HS278	0.479	0.558	26.93%	0.505	125	0.498	124	0.469	150	0.503	0.034	93.30%
HS279	0.419	0.326	34.47%	0.393	284	0.384	285	0.392	303	0.344	0.048	85.99%
HS280	0.290	0.394	37.79%	0.323	376	0.319	372	0.320	404	0.351	0.031	91.19%
HS281	0.496	0.412	48.41%	0.510	116	0.502	118	0.478	138	0.429	0.050	88.45%
HS282	0.387	0.663	42.55%	0.538	90	0.536	79	0.444	197	0.435	0.009	98.00%
HS283	0.408	0.219	20.11%	0.287	411	0.279	412	0.348	373	0.351	0.004	98.87%
HS284	0.257	0.403	29.22%	0.296	402	0.293	399	0.288	433	0.299	0.011	96.34%
HS285	0.113	0.224	37.91%	0.126	491	0.128	490	0.165	491	0.183	0.018	90.27%
HS286	0.235	0.591	45.26%	0.398	275	0.400	262	0.328	395	0.361	0.032	91.04%
HS287	0.440	0.162	34.84%	0.306	393	0.298	394	0.376	329	0.387	0.010	97.29%
HS288	0.315	0.372	52.49%	0.352	335	0.349	330	0.351	367	0.367	0.016	95.57%
HS289	0.270	0.353	49.93%	0.330	369	0.327	367	0.313	411	0.273	0.040	85.23%
HS290	0.373	0.347	44.44%	0.388	293	0.382	288	0.377	328	0.335	0.042	87.41%
HS291	0.359	0.323	67.65%	0.384	299	0.381	293	0.390	310	0.427	0.037	91.30%
HS292	0.476	0.171	42.95%	0.353	334	0.342	339	0.413	263	0.403	0.010	97.64%
HS293	0.570	0.239	39.40%	0.462	181	0.448	188	0.487	122	0.423	0.063	85.06%
HS294	0.344	0.383	58.85%	0.397	280	0.394	268	0.380	324	0.384	0.004	99.08%
HS295	0.408	0.278	56.51%	0.373	314	0.368	308	0.402	282	0.425	0.023	94.70%
HS296	0.302	0.291	34.07%	0.263	429	0.259	430	0.304	418	0.356	0.051	85.56%
HS297	0.277	0.588	64.16%	0.443	211	0.447	194	0.379	326	0.438	0.060	86.42%
HS298	0.445	0.393	51.53%	0.444	208	0.439	206	0.444	198	0.460	0.017	96.37%
HS299	0.353	0.425	27.07%	0.377	310	0.368	307	0.357	358	0.331	0.026	92.20%
HS300	0.384	0.375	40.42%	0.383	301	0.376	299	0.385	317	0.398	0.013	96.73%
HS301	0.323	0.304	44.82%	0.314	387	0.309	385	0.334	388	0.345	0.011	96.95%
HS302	0.433	0.392	61.77%	0.460	184	0.455	177	0.447	188	0.465	0.018	96.07%
HS303	0.414	0.429	34.91%	0.422	237	0.413	236	0.409	269	0.412	0.003	99.32%

HS304	0.464	0.521	15.55%	0.471	166	0.462	168	0.438	209	0.427	0.011	97.41%
HS305	0.706	0.308	38.51%	0.579	47	0.562	53	0.592	24	0.534	0.059	88.97%
HS306	0.530	0.481	42.08%	0.554	71	0.544	69	0.508	96	0.458	0.050	89.13%
HS307	0.200	0.276	31.51%	0.197	470	0.196	468	0.228	468	0.247	0.019	92.43%
HS308	0.676	0.180	47.11%	0.510	118	0.492	131	0.558	47	0.505	0.053	89.55%
HS309	0.243	0.225	43.84%	0.233	447	0.230	449	0.263	454	0.254	0.009	96.55%
HS310	0.380	0.326	35.28%	0.350	341	0.342	340	0.367	344	0.359	0.008	97.85%
HS311	0.400	0.233	41.59%	0.329	370	0.321	371	0.370	339	0.371	0.000	99.90%
HS312	0.271	0.194	44.11%	0.225	455	0.222	453	0.277	448	0.295	0.018	93.85%
HS313	0.493	0.300	40.96%	0.428	230	0.418	232	0.446	191	0.424	0.022	94.74%
HS314	0.334	0.149	36.91%	0.225	456	0.220	455	0.303	420	0.345	0.042	87.74%
HS315	0.287	0.198	30.23%	0.215	459	0.209	460	0.272	451	0.286	0.014	95.17%
HS316	0.764	0.268	43.95%	0.616	25	0.596	31	0.632	11	0.544	0.088	83.78%
HS317	0.432	0.574	39.28%	0.518	107	0.512	105	0.455	170	0.462	0.007	98.43%
HS318	0.603	0.377	40.94%	0.561	63	0.545	68	0.537	67	0.447	0.090	79.88%
HS319	0.351	0.393	92.20%	0.466	172	0.467	163	0.427	233	0.443	0.016	96.42%
HS320	0.672	0.374	59.54%	0.621	24	0.610	24	0.607	15	0.570	0.036	93.61%
HS321	0.683	0.227	70.27%	0.597	36	0.581	41	0.599	19	0.478	0.121	74.65%
HS322	0.493	0.363	54.33%	0.498	131	0.490	133	0.475	145	0.417	0.057	86.28%
HS323	0.380	0.392	36.19%	0.390	286	0.383	287	0.380	325	0.359	0.020	94.31%
HS324	0.585	0.285	34.27%	0.480	159	0.467	162	0.499	104	0.447	0.052	88.26%
HS325	0.631	0.359	84.21%	0.645	13	0.636	13	0.605	16	0.535	0.070	86.93%
HS326	0.499	0.231	30.08%	0.377	309	0.366	310	0.425	238	0.406	0.019	95.40%
HS327	0.543	0.184	34.44%	0.376	311	0.365	312	0.452	181	0.480	0.028	94.13%
HS328	0.607	0.474	37.18%	0.560	66	0.551	62	0.554	53	0.608	0.054	91.11%
HS329	0.530	0.217	37.69%	0.403	267	0.391	274	0.452	178	0.439	0.014	96.81%
HS330	0.639	0.442	58.76%	0.615	26	0.608	25	0.596	21	0.613	0.017	97.27%
HS331	0.814	0.620	47.75%	0.778	3	0.770	3	0.737	3	0.818	0.080	90.20%

HS332	0.670	0.299	43.96%	0.542	80	0.528	90	0.573	36	0.570	0.003	99.43%
HS333	0.631	0.263	34.42%	0.476	161	0.463	167	0.527	75	0.542	0.015	97.28%
HS334	0.695	0.269	40.37%	0.539	89	0.524	93	0.580	31	0.563	0.017	97.05%
HS335	0.696	0.223	36.52%	0.514	113	0.498	125	0.567	41	0.539	0.028	94.87%
HS336	0.920	0.337	29.48%	0.681	10	0.666	10	0.736	5	0.776	0.040	94.84%
HS337	0.626	0.401	41.80%	0.562	61	0.551	63	0.559	46	0.555	0.004	99.37%
HS338	0.528	0.308	46.09%	0.457	190	0.448	190	0.479	136	0.492	0.013	97.41%
HS339	0.329	0.308	40.30%	0.309	390	0.305	391	0.334	387	0.364	0.030	91.76%
HS340	0.323	0.317	42.19%	0.316	383	0.313	379	0.333	389	0.365	0.031	91.46%
HS341	0.678	0.284	38.38%	0.540	86	0.525	92	0.568	40	0.544	0.025	95.48%
HS342	0.284	0.211	36.23%	0.225	454	0.220	456	0.279	444	0.310	0.030	90.21%
HS343	0.491	0.336	30.85%	0.431	227	0.420	231	0.440	205	0.408	0.032	92.26%
HS344	0.387	0.371	39.03%	0.379	305	0.371	304	0.384	318	0.397	0.013	96.78%
HS345	0.525	0.373	40.28%	0.484	157	0.473	156	0.482	130	0.470	0.012	97.48%
HS346	0.531	0.213	31.87%	0.407	261	0.393	271	0.446	193	0.382	0.064	83.10%
HS347	0.348	0.242	32.47%	0.279	417	0.272	419	0.325	399	0.344	0.019	94.60%
HS348	0.381	0.223	30.71%	0.298	400	0.288	402	0.343	377	0.329	0.013	95.98%
HS349	0.324	0.519	30.26%	0.414	249	0.410	243	0.358	357	0.333	0.025	92.39%
HS350	0.497	0.322	41.53%	0.464	175	0.453	183	0.454	172	0.383	0.071	81.43%
HS351	0.381	0.379	39.22%	0.383	302	0.377	297	0.382	323	0.395	0.013	96.60%
HS352	0.524	0.440	36.91%	0.495	136	0.489	134	0.490	118	0.522	0.032	93.89%
HS353	0.634	0.340	28.19%	0.532	93	0.518	98	0.537	68	0.481	0.055	88.47%
HS354	0.445	0.454	36.19%	0.465	174	0.458	171	0.437	212	0.423	0.014	96.71%
HS355	0.494	0.265	37.24%	0.401	269	0.391	273	0.436	215	0.427	0.009	97.90%
HS356	0.551	0.193	46.34%	0.435	219	0.422	229	0.473	146	0.426	0.047	89.03%
HS357	0.478	0.341	36.61%	0.437	215	0.429	218	0.439	207	0.404	0.035	91.22%
HS358	0.488	0.341	27.78%	0.448	204	0.435	211	0.435	217	0.345	0.091	73.75%
HS359	0.300	0.294	30.58%	0.274	422	0.269	421	0.300	425	0.313	0.014	95.59%

HS360	0.470	0.303	27.70%	0.411	259	0.399	264	0.416	258	0.350	0.065	81.36%
HS361	0.283	0.441	37.23%	0.350	338	0.346	336	0.323	401	0.318	0.005	98.34%
HS362	0.361	0.301	40.13%	0.338	362	0.332	356	0.355	361	0.353	0.002	99.55%
HS363	0.345	0.395	51.64%	0.400	272	0.396	266	0.375	332	0.369	0.006	98.31%
HS364	0.215	0.463	35.84%	0.308	391	0.308	387	0.279	445	0.316	0.037	88.17%
HS365	0.345	0.257	27.28%	0.289	407	0.281	409	0.320	405	0.301	0.019	93.68%
HS366	0.550	0.510	26.98%	0.560	64	0.553	61	0.509	93	0.460	0.049	89.42%
HS367	0.416	0.245	40.81%	0.355	329	0.348	333	0.383	321	0.358	0.025	93.08%
HS368	0.624	0.454	31.69%	0.597	37	0.583	40	0.555	51	0.474	0.081	82.92%
HS369	0.384	0.531	40.50%	0.487	148	0.481	147	0.415	259	0.359	0.055	84.62%
HS370	0.447	0.299	29.42%	0.372	316	0.362	315	0.401	286	0.400	0.000	99.90%
HS371	0.464	0.209	33.81%	0.348	344	0.338	346	0.401	287	0.402	0.002	99.62%
HS372	0.658	0.268	41.28%	0.530	95	0.515	103	0.555	52	0.500	0.054	89.14%
HS373	0.463	0.200	33.47%	0.349	342	0.338	347	0.398	290	0.370	0.029	92.27%
HS374	0.531	0.296	43.87%	0.465	173	0.454	179	0.476	141	0.443	0.033	92.45%
HS375	0.266	0.269	51.87%	0.275	421	0.273	417	0.297	426	0.327	0.031	90.65%
HS376	0.303	0.528	33.18%	0.389	287	0.387	283	0.349	371	0.386	0.037	90.42%
HS377	0.432	0.438	35.25%	0.460	183	0.454	178	0.423	241	0.377	0.046	87.70%
HS378	0.419	0.554	41.62%	0.495	137	0.491	132	0.444	196	0.469	0.024	94.79%
HS379	0.580	0.355	32.40%	0.498	133	0.488	136	0.507	99	0.491	0.016	96.65%
HS380	0.625	0.060	45.75%	0.415	246	0.401	261	0.498	106	0.450	0.049	89.19%
HS381	0.483	0.300	40.07%	0.432	225	0.422	228	0.439	208	0.391	0.047	87.88%
HS382	0.287	0.269	30.47%	0.245	439	0.240	440	0.285	435	0.315	0.029	90.67%
HS383	0.553	0.332	35.12%	0.457	189	0.448	191	0.487	121	0.521	0.034	93.45%
HS384	0.344	0.156	40.03%	0.254	436	0.248	436	0.315	410	0.313	0.002	99.41%
HS385	0.478	0.275	33.97%	0.381	304	0.372	303	0.423	245	0.436	0.013	96.97%
HS386	0.590	0.364	32.63%	0.484	155	0.473	157	0.516	87	0.559	0.043	92.31%
HS387	0.260	0.268	32.06%	0.243	441	0.238	441	0.269	453	0.261	0.007	97.23%

HS388	0.107	0.255	19.87%	0.102	495	0.102	495	0.146	495	0.188	0.042	77.55%
HS389	0.325	0.135	24.17%	0.213	460	0.204	464	0.279	443	0.244	0.036	85.29%
HS390	0.433	0.214	20.75%	0.305	396	0.293	398	0.365	348	0.360	0.005	98.57%
HS391	0.141	0.271	31.11%	0.150	483	0.149	484	0.186	488	0.231	0.045	80.41%
HS392	0.136	0.287	39.08%	0.177	479	0.177	477	0.195	484	0.209	0.014	93.33%
HS393	0.516	0.252	42.84%	0.422	236	0.413	238	0.456	168	0.441	0.015	96.48%
HS394	0.331	0.323	28.80%	0.292	405	0.286	405	0.324	400	0.366	0.042	88.46%
HS395	0.511	0.506	42.22%	0.529	96	0.522	94	0.499	105	0.534	0.035	93.47%
HS396	0.384	0.356	42.69%	0.384	300	0.378	296	0.383	320	0.383	0.000	99.94%
HS397	0.310	0.420	88.97%	0.444	209	0.447	192	0.400	289	0.429	0.029	93.17%
HS398	0.456	0.229	46.78%	0.372	315	0.363	314	0.414	260	0.423	0.008	98.08%
HS399	0.464	0.178	36.36%	0.324	373	0.313	380	0.398	291	0.434	0.035	91.82%
HS400	0.443	0.377	37.40%	0.419	241	0.411	239	0.422	248	0.432	0.009	97.83%
HS401	0.281	0.374	39.22%	0.314	386	0.311	383	0.312	413	0.332	0.020	93.97%
HS402	0.648	0.249	22.68%	0.488	147	0.471	158	0.522	80	0.450	0.072	83.98%
HS403	0.362	0.403	63.69%	0.433	224	0.428	219	0.403	278	0.393	0.010	97.53%
HS404	0.708	0.306	38.37%	0.563	60	0.549	67	0.594	23	0.579	0.015	97.46%
HS405	0.458	0.347	59.38%	0.449	203	0.442	203	0.453	176	0.467	0.014	97.08%
HS406	0.661	0.385	21.17%	0.523	103	0.512	104	0.556	50	0.606	0.051	91.66%
HS407	0.320	0.284	48.27%	0.307	392	0.303	393	0.333	390	0.350	0.018	94.88%
HS408	0.538	0.415	36.25%	0.498	130	0.488	135	0.494	113	0.506	0.013	97.53%
HS409	0.454	0.355	40.69%	0.430	229	0.422	230	0.430	228	0.421	0.008	98.02%
HS410	0.338	0.343	32.82%	0.314	388	0.307	389	0.338	382	0.384	0.046	87.96%
HS411	0.545	0.286	40.25%	0.468	169	0.455	176	0.479	133	0.412	0.067	83.74%
HS412	0.409	0.326	38.63%	0.385	296	0.378	295	0.391	308	0.382	0.009	97.66%
HS413	0.426	0.362	28.69%	0.389	289	0.381	291	0.397	292	0.393	0.004	98.89%
HS414	0.353	0.301	40.63%	0.336	365	0.331	360	0.350	369	0.353	0.003	99.04%
HS415	0.192	0.354	32.88%	0.223	457	0.220	454	0.239	464	0.289	0.050	82.67%

HS416	0.422	0.286	38.13%	0.367	318	0.359	317	0.392	306	0.383	0.008	97.80%
HS417	0.459	0.226	28.63%	0.341	356	0.329	364	0.395	299	0.409	0.014	96.57%
HS418	0.565	0.336	30.52%	0.491	143	0.477	154	0.491	116	0.421	0.069	83.53%
HS419	0.397	0.413	36.81%	0.409	260	0.404	257	0.397	293	0.403	0.006	98.44%
HS420	0.317	0.264	29.34%	0.277	419	0.270	420	0.304	419	0.283	0.021	92.73%
HS421	0.484	0.390	36.41%	0.464	177	0.454	180	0.452	180	0.422	0.029	93.05%
HS422	0.584	0.182	21.74%	0.397	281	0.382	290	0.464	157	0.440	0.024	94.54%
HS423	0.367	0.164	39.44%	0.284	414	0.275	416	0.332	391	0.303	0.029	90.55%
HS424	0.337	0.339	48.30%	0.355	330	0.350	329	0.355	360	0.352	0.002	99.35%
HS425	0.495	0.224	29.08%	0.364	321	0.353	326	0.419	252	0.427	0.007	98.31%
HS426	0.529	0.632	17.86%	0.584	45	0.574	45	0.507	100	0.475	0.032	93.24%
HS427	0.317	0.330	47.00%	0.341	357	0.336	350	0.338	383	0.326	0.012	96.45%
HS428	0.343	0.288	35.79%	0.315	384	0.308	386	0.334	386	0.318	0.016	95.00%
HS429	0.583	0.256	71.33%	0.525	101	0.517	99	0.537	69	0.507	0.030	94.06%
HS430	0.538	0.354	50.98%	0.503	128	0.494	128	0.500	103	0.486	0.014	97.07%
HS431	0.663	0.232	39.19%	0.508	120	0.493	130	0.549	57	0.511	0.038	92.50%
HS432	0.370	0.588	46.69%	0.503	127	0.501	121	0.423	244	0.414	0.009	97.71%
HS433	0.487	0.282	46.66%	0.413	254	0.406	251	0.446	194	0.470	0.025	94.79%
HS434	0.374	0.559	38.56%	0.462	180	0.458	172	0.411	267	0.450	0.039	91.28%
HS435	0.554	0.339	32.73%	0.463	179	0.453	182	0.486	123	0.483	0.003	99.40%
HS436	0.667	0.319	32.55%	0.543	79	0.528	89	0.560	45	0.510	0.051	90.02%
HS437	0.817	0.275	40.14%	0.631	18	0.613	23	0.665	10	0.618	0.048	92.29%
HS438	0.449	0.447	44.15%	0.489	146	0.482	146	0.448	187	0.417	0.031	92.63%
HS439	0.492	0.576	39.92%	0.556	69	0.549	66	0.497	107	0.507	0.010	98.00%
HS440	0.584	0.684	31.83%	0.638	16	0.633	15	0.571	37	0.629	0.058	90.76%
HS441	0.393	0.530	33.47%	0.456	192	0.451	186	0.412	265	0.425	0.014	96.77%
HS442	0.515	0.662	46.77%	0.603	32	0.599	28	0.537	66	0.606	0.069	88.60%
HS443	0.355	0.541	35.29%	0.430	228	0.427	220	0.390	309	0.435	0.044	89.78%

HS444	0.409	0.731	40.44%	0.564	59	0.565	51	0.469	149	0.523	0.053	89.85%
HS445	0.321	0.303	32.74%	0.288	408	0.283	408	0.319	406	0.359	0.040	88.82%
HS446	1.000	0.232	41.53%	0.749	5	0.725	5	0.785	1	0.670	0.115	82.78%
HS447	0.832	0.594	48.96%	0.804	2	0.792	2	0.747	2	0.742	0.005	99.36%
HS448	0.576	0.487	41.14%	0.571	52	0.561	54	0.540	65	0.521	0.019	96.36%
HS449	0.461	0.718	45.90%	0.621	23	0.618	22	0.509	92	0.513	0.004	99.26%
HS450	0.400	0.658	41.01%	0.532	94	0.530	85	0.450	182	0.480	0.029	93.85%
HS451	0.389	0.566	42.21%	0.498	132	0.494	129	0.426	234	0.413	0.013	96.78%
HS452	0.316	0.371	67.44%	0.388	292	0.388	279	0.369	342	0.388	0.019	95.10%
HS453	0.480	0.585	35.73%	0.540	81	0.534	81	0.485	125	0.511	0.026	94.99%
HS454	0.413	0.393	36.95%	0.413	256	0.406	249	0.404	275	0.405	0.001	99.72%
HS455	0.615	0.575	36.76%	0.640	15	0.630	16	0.578	33	0.547	0.031	94.33%
HS456	0.317	0.476	36.32%	0.382	303	0.379	294	0.353	364	0.380	0.027	92.93%
HS457	0.479	0.655	46.12%	0.595	38	0.591	34	0.510	90	0.525	0.014	97.25%
HS458	0.507	0.647	47.45%	0.628	22	0.621	20	0.529	73	0.498	0.032	93.59%
HS459	0.570	0.330	33.61%	0.475	163	0.463	166	0.497	108	0.477	0.020	95.83%
HS460	0.375	0.630	31.71%	0.501	129	0.496	127	0.417	255	0.393	0.024	93.91%
HS461	0.542	0.600	50.78%	0.607	29	0.604	26	0.549	58	0.609	0.060	90.15%
HS462	0.328	0.511	40.63%	0.414	252	0.410	241	0.372	337	0.392	0.020	94.86%
HS463	0.414	0.520	40.80%	0.492	142	0.487	138	0.434	218	0.423	0.010	97.55%
HS464	0.576	0.462	48.94%	0.597	35	0.586	37	0.544	61	0.478	0.066	86.09%
HS465	0.461	0.511	44.11%	0.507	121	0.501	120	0.468	151	0.487	0.019	96.02%
HS466	0.374	0.529	44.37%	0.468	170	0.465	164	0.411	266	0.417	0.006	98.63%
HS467	0.151	0.792	40.72%	0.426	231	0.432	214	0.302	421	0.357	0.054	84.76%
HS468	0.371	0.573	48.17%	0.487	151	0.483	142	0.422	249	0.434	0.012	97.19%
HS469	0.441	0.578	41.36%	0.539	87	0.533	82	0.463	159	0.429	0.034	92.08%
HS470	0.443	0.593	37.13%	0.526	99	0.522	95	0.463	161	0.470	0.007	98.47%
HS471	0.802	0.477	40.78%	0.764	4	0.744	4	0.694	7	0.534	0.160	70.02%

HS472	0.378	0.694	49.71%	0.561	62	0.559	57	0.452	179	0.451	0.001	99.87%
HS473	0.358	0.401	43.91%	0.398	278	0.394	269	0.376	331	0.365	0.011	97.01%
HS474	0.347	0.610	38.23%	0.483	158	0.480	149	0.401	285	0.386	0.015	96.05%
HS475	0.362	0.601	49.93%	0.496	135	0.497	126	0.423	243	0.465	0.042	90.96%
HS476	0.348	0.905	52.90%	0.646	12	0.649	12	0.475	143	0.488	0.013	97.37%
HS477	0.353	0.682	36.19%	0.514	114	0.510	108	0.417	256	0.415	0.002	99.63%
HS478	0.553	0.634	44.53%	0.643	14	0.635	14	0.556	49	0.528	0.028	94.76%
HS479	0.490	0.589	35.03%	0.568	56	0.560	56	0.492	114	0.455	0.037	91.80%
HS480	0.714	0.350	34.84%	0.613	27	0.596	32	0.602	17	0.506	0.096	80.99%
HS481	0.514	0.475	40.64%	0.551	73	0.540	75	0.494	111	0.418	0.076	81.69%
HS482	0.353	0.601	37.32%	0.489	145	0.484	140	0.402	283	0.363	0.039	89.38%
HS483	0.524	0.247	43.60%	0.433	223	0.423	226	0.461	164	0.423	0.038	91.01%
HS484	0.371	0.204	34.22%	0.287	410	0.279	413	0.336	384	0.326	0.010	97.08%
HS485	0.347	0.315	38.77%	0.347	348	0.340	344	0.346	374	0.302	0.044	85.44%
HS486	0.556	0.087	43.36%	0.364	322	0.353	325	0.453	177	0.446	0.007	98.53%
HS487	0.662	0.149	46.50%	0.494	140	0.478	152	0.541	64	0.462	0.079	82.79%
HS488	0.476	0.504	24.02%	0.495	138	0.487	137	0.453	174	0.426	0.028	93.53%
HS489	0.149	0.235	58.88%	0.199	467	0.200	465	0.218	475	0.230	0.013	94.50%
HS490	0.094	0.256	32.04%	0.125	492	0.125	491	0.152	493	0.158	0.006	96.10%
HS491	0.217	0.178	16.44%	0.143	485	0.136	485	0.203	480	0.194	0.009	95.17%
HS492	0.322	0.737	23.09%	0.515	112	0.512	107	0.390	311	0.348	0.042	88.00%
HS493	0.532	0.274	35.10%	0.435	217	0.423	225	0.462	163	0.427	0.035	91.87%
HS494	0.360	0.503	26.83%	0.421	238	0.414	235	0.376	330	0.348	0.028	92.04%
HS495	0.296	0.179	43.01%	0.236	446	0.232	448	0.290	431	0.297	0.007	97.52%
HS496	0.191	0.199	46.76%	0.197	469	0.195	469	0.226	470	0.197	0.029	85.31%
HS497	0.575	0.300	29.53%	0.459	186	0.447	193	0.489	119	0.478	0.011	97.67%
HS498	0.502	0.494	32.56%	0.516	111	0.507	111	0.479	132	0.463	0.017	96.35%
HS499	0.469	0.155	58.52%	0.384	298	0.375	300	0.423	242	0.371	0.052	85.87%

HS500	0.425	0.314	69.84%	0.434	221	0.427	221	0.437	213	0.445	0.008	98.15%
HS501	0.291	0.266	23.91%	0.243	442	0.235	444	0.280	442	0.280	0.000	99.94%
HS502	0.390	0.201	25.98%	0.286	413	0.276	415	0.339	381	0.327	0.012	96.42%
Av				0.409		0.402		0.413				92.98%
CV				33.27%		33.40%		27.88%				

Table S2. D-value, WDC-value and CDC-value of REP 2 in 502 germplasm resources

Code	REP 2											
	Subordinate function value			WDC-value	Rank	CDC-value	Rank	D-value	Rank	Predicted D-value	Absolute value of error	forecast accuracy
	μ1	μ2	μ3									
HS1	0.511	0.627	0.095	0.600	68	0.632	64	0.496	94	0.456	0.040	91.23%
HS2	0.225	0.399	0.117	0.343	364	0.359	364	0.276	390	0.264	0.012	95.45%
HS3	0.545	0.656	0.043	0.614	55	0.633	62	0.515	70	0.544	0.029	94.67%
HS4	0.690	0.639	0.092	0.689	12	0.714	12	0.584	17	0.607	0.023	96.21%
HS5	0.462	0.708	0.093	0.606	62	0.620	76	0.504	78	0.538	0.034	93.68%
HS6	0.305	0.365	0.150	0.372	328	0.396	324	0.306	344	0.295	0.011	96.27%
HS7	0.663	0.651	0.160	0.691	10	0.719	11	0.586	14	0.581	0.005	99.14%
HS8	0.741	0.304	0.191	0.560	104	0.583	112	0.494	96	0.528	0.034	93.56%
HS9	0.854	0.429	0.067	0.665	21	0.685	25	0.577	23	0.588	0.011	98.13%
HS10	0.752	0.590	0.120	0.691	11	0.708	14	0.598	10	0.684	0.086	87.43%
HS11	0.897	0.266	0.156	0.619	49	0.653	49	0.547	40	0.554	0.007	98.74%
HS12	0.907	0.349	0.067	0.651	30	0.679	28	0.571	25	0.582	0.011	98.11%
HS13	0.794	0.377	0.265	0.638	34	0.674	32	0.557	30	0.535	0.022	95.89%
HS14	0.494	0.193	0.210	0.384	317	0.408	313	0.337	304	0.348	0.011	96.84%
HS15	0.642	0.271	0.108	0.483	203	0.508	204	0.422	196	0.478	0.056	88.28%
HS16	0.845	0.017	0.170	0.477	209	0.516	196	0.429	182	0.395	0.034	91.39%
HS17	0.712	0.171	0.101	0.472	215	0.498	218	0.416	206	0.439	0.023	94.76%
HS18	0.720	0.336	0.194	0.565	99	0.588	106	0.497	92	0.529	0.032	93.95%
HS19	0.738	0.284	0.157	0.549	119	0.580	116	0.479	109	0.463	0.016	96.54%
HS20	0.760	0.484	0.116	0.656	26	0.690	21	0.561	28	0.489	0.072	85.28%
HS21	0.691	0.351	0.088	0.546	122	0.568	128	0.473	118	0.526	0.053	89.92%
HS22	0.829	0.265	0.167	0.587	77	0.622	75	0.517	68	0.514	0.003	99.42%
HS23	0.975	0.079	0.062	0.562	101	0.605	87	0.499	86	0.449	0.050	88.86%

HS24	0.665	0.376	0.089	0.550	116	0.578	119	0.471	123	0.449	0.022	95.10%
HS25	0.594	0.439	0.187	0.558	107	0.585	108	0.476	114	0.453	0.023	94.92%
HS26	0.634	0.394	0.122	0.548	120	0.576	121	0.468	128	0.481	0.013	97.30%
HS27	0.715	0.309	0.096	0.537	130	0.558	141	0.47	124	0.504	0.034	93.25%
HS28	0.674	0.439	0.083	0.581	81	0.602	89	0.498	91	0.529	0.031	94.14%
HS29	0.559	0.516	0.132	0.571	92	0.598	96	0.481	107	0.492	0.011	97.76%
HS30	0.819	0.242	0.167	0.576	88	0.619	78	0.503	80	0.453	0.050	88.96%
HS31	0.592	0.631	0.238	0.655	27	0.682	27	0.556	32	0.567	0.011	98.06%
HS32	0.560	0.348	0.048	0.477	208	0.504	213	0.405	220	0.449	0.044	90.20%
HS33	0.558	0.395	0.094	0.505	170	0.532	174	0.429	182	0.416	0.013	96.88%
HS34	0.847	0.191	0.204	0.563	100	0.595	100	0.502	82	0.492	0.010	97.97%
HS35	0.637	0.295	0.122	0.496	181	0.520	191	0.431	178	0.457	0.026	94.31%
HS36	0.768	0.351	0.229	0.605	65	0.634	59	0.53	54	0.499	0.031	93.79%
HS37	0.926	0.161	0.138	0.581	82	0.617	79	0.518	67	0.504	0.014	97.22%
HS38	0.815	0.202	0.115	0.545	125	0.578	118	0.478	112	0.423	0.055	87.00%
HS39	0.503	0.311	0.222	0.450	240	0.475	242	0.389	239	0.389	0.000	100.00%
HS40	0.647	0.231	0.199	0.487	194	0.524	186	0.423	188	0.381	0.042	88.98%
HS41	0.417	0.313	0.175	0.403	299	0.426	295	0.342	302	0.362	0.020	94.48%
HS42	0.441	0.736	0.232	0.628	44	0.646	52	0.524	61	0.552	0.028	94.93%
HS43	0.639	0.309	0.084	0.504	172	0.533	172	0.432	175	0.418	0.014	96.65%
HS44	0.682	0.511	0.179	0.637	39	0.663	38	0.544	42	0.501	0.043	91.42%
HS45	0.479	0.750	0.101	0.638	33	0.653	48	0.529	55	0.606	0.077	87.29%
HS46	0.729	0.265	0.024	0.520	153	0.547	151	0.449	152	0.443	0.006	98.65%
HS47	0.845	0.161	0.188	0.546	123	0.581	115	0.487	101	0.494	0.007	98.58%
HS48	0.871	0.226	0.213	0.596	72	0.629	68	0.528	56	0.507	0.021	95.86%
HS49	0.791	0.153	0.173	0.512	161	0.542	158	0.456	144	0.449	0.007	98.44%
HS50	0.566	0.612	0.284	0.637	36	0.660	40	0.543	43	0.598	0.055	90.80%
HS51	0.689	0.290	0.180	0.533	134	0.568	129	0.462	134	0.433	0.029	93.30%

HS52	0.712	0.462	0.130	0.630	42	0.666	36	0.532	53	0.436	0.096	77.98%
HS53	0.501	0.357	0.136	0.461	225	0.485	230	0.393	233	0.406	0.013	96.80%
HS54	0.828	0.329	0.142	0.617	51	0.655	46	0.537	48	0.512	0.025	95.12%
HS55	0.679	0.174	0.155	0.468	220	0.499	216	0.409	216	0.363	0.046	87.33%
HS56	0.620	0.347	0.089	0.511	163	0.537	165	0.438	163	0.434	0.004	99.08%
HS57	0.616	0.400	0.051	0.530	137	0.554	146	0.452	148	0.472	0.020	95.76%
HS58	1.000	0.159	0.121	0.619	48	0.659	41	0.55	36	0.499	0.051	89.78%
HS59	0.527	0.293	0.053	0.437	259	0.460	264	0.369	267	0.339	0.030	91.15%
HS60	0.567	0.104	0.086	0.367	334	0.393	326	0.32	324	0.287	0.033	88.50%
HS61	0.428	0.098	0.043	0.288	436	0.309	432	0.246	436	0.24	0.006	97.50%
HS62	0.655	0.061	0.379	0.422	279	0.453	271	0.387	243	0.378	0.009	97.62%
HS63	0.519	0.351	0.133	0.467	222	0.488	225	0.399	227	0.414	0.015	96.38%
HS64	0.827	0.082	0.045	0.478	207	0.508	207	0.428	184	0.428	0.000	100.00%
HS65	0.762	0.347	0.096	0.578	85	0.597	97	0.506	77	0.527	0.021	96.02%
HS66	0.712	0.230	0.086	0.492	188	0.508	205	0.436	167	0.47	0.034	92.77%
HS67	0.594	0.329	0.101	0.487	193	0.509	203	0.421	199	0.461	0.040	91.32%
HS68	0.866	0.369	0.226	0.657	25	0.675	30	0.583	18	0.607	0.024	96.05%
HS69	0.731	0.322	0.246	0.583	79	0.627	69	0.504	78	0.432	0.072	83.33%
HS70	0.696	0.258	0.223	0.525	144	0.561	139	0.459	140	0.439	0.020	95.44%
HS71	0.678	0.207	0.155	0.487	195	0.525	185	0.421	199	0.378	0.043	88.62%
HS72	0.676	0.342	0.117	0.554	111	0.598	94	0.467	131	0.377	0.090	76.13%
HS73	0.558	0.472	0.168	0.557	108	0.592	102	0.469	127	0.408	0.061	85.05%
HS74	0.522	0.232	0.093	0.411	288	0.448	280	0.349	295	0.293	0.056	80.89%
HS75	0.586	0.417	0.039	0.529	141	0.566	133	0.442	159	0.394	0.048	87.82%
HS76	0.546	0.601	0.168	0.618	50	0.659	42	0.513	73	0.384	0.129	66.41%
HS77	0.637	0.240	0.164	0.482	206	0.521	189	0.416	206	0.385	0.031	91.95%
HS78	0.459	0.436	0.158	0.484	202	0.512	202	0.407	219	0.391	0.016	95.91%
HS79	0.787	0.163	0.091	0.516	156	0.566	132	0.447	155	0.381	0.066	82.68%

HS80	0.441	0.235	0.133	0.375	325	0.404	318	0.317	330	0.265	0.052	80.38%
HS81	0.650	0.342	0.313	0.557	109	0.596	98	0.483	104	0.442	0.041	90.72%
HS82	0.508	0.207	0.124	0.396	305	0.427	294	0.337	304	0.308	0.029	90.58%
HS83	0.414	0.410	0.152	0.450	241	0.476	241	0.375	262	0.365	0.010	97.26%
HS84	0.450	0.325	0.185	0.429	270	0.455	270	0.364	273	0.356	0.008	97.75%
HS85	0.242	0.440	0.151	0.374	327	0.390	332	0.305	347	0.307	0.002	99.35%
HS86	0.318	0.273	0.130	0.326	389	0.343	388	0.273	395	0.299	0.026	91.30%
HS87	0.362	0.211	0.067	0.312	412	0.333	409	0.261	420	0.273	0.012	95.60%
HS88	0.225	0.354	0.285	0.339	373	0.355	371	0.284	379	0.302	0.018	94.04%
HS89	0.313	0.166	0.155	0.277	452	0.299	442	0.234	451	0.227	0.007	96.92%
HS90	0.583	0.585	0.148	0.622	47	0.658	45	0.521	66	0.484	0.037	92.36%
HS91	0.866	0.365	0.205	0.659	23	0.687	22	0.578	22	0.556	0.022	96.04%
HS92	0.690	0.334	0.176	0.550	117	0.575	122	0.479	109	0.494	0.015	96.96%
HS93	0.737	0.270	0.157	0.536	133	0.558	143	0.474	117	0.514	0.040	92.22%
HS94	0.732	0.551	0.198	0.676	15	0.692	18	0.586	14	0.638	0.052	91.85%
HS95	0.665	0.404	0.214	0.576	87	0.602	90	0.499	86	0.496	0.003	99.40%
HS96	0.462	0.673	0.095	0.592	76	0.614	81	0.49	99	0.481	0.009	98.13%
HS97	0.719	0.328	0.101	0.554	112	0.582	113	0.479	109	0.481	0.002	99.58%
HS98	0.688	0.406	0.128	0.580	84	0.607	85	0.499	86	0.496	0.003	99.40%
HS99	0.323	0.288	0.122	0.335	376	0.353	375	0.281	382	0.304	0.023	92.43%
HS100	0.724	0.147	0.159	0.476	212	0.508	209	0.421	199	0.417	0.004	99.04%
HS101	0.618	0.286	0.048	0.475	213	0.498	217	0.408	218	0.421	0.013	96.91%
HS102	0.802	0.060	0.081	0.466	223	0.507	210	0.413	211	0.367	0.046	87.47%
HS103	0.369	0.591	0.153	0.509	166	0.526	182	0.423	188	0.439	0.016	96.36%
HS104	0.855	0.324	0.100	0.614	56	0.634	60	0.542	46	0.583	0.041	92.97%
HS105	0.776	0.153	0.054	0.497	180	0.533	170	0.432	175	0.378	0.054	85.71%
HS106	0.600	0.509	0.146	0.592	75	0.624	71	0.5	85	0.486	0.014	97.12%
HS107	0.617	0.210	0.098	0.446	246	0.477	240	0.386	245	0.343	0.043	87.46%

HS108	0.626	0.366	0.122	0.523	146	0.548	149	0.453	147	0.5	0.047	90.60%
HS109	0.704	0.212	0.113	0.491	189	0.518	194	0.43	180	0.426	0.004	99.06%
HS110	0.670	0.185	0.079	0.457	228	0.483	234	0.398	229	0.385	0.013	96.62%
HS111	0.691	0.302	0.076	0.523	148	0.547	152	0.452	148	0.465	0.013	97.20%
HS112	0.767	0.416	0.031	0.613	57	0.636	56	0.526	59	0.523	0.003	99.43%
HS113	0.689	0.559	0.691	0.719	7	0.748	6	0.639	4	0.613	0.026	95.76%
HS114	0.610	0.390	0.095	0.530	139	0.558	142	0.451	150	0.428	0.023	94.63%
HS115	0.678	0.616	0.134	0.673	16	0.699	17	0.576	24	0.589	0.013	97.79%
HS116	0.704	0.194	0.096	0.485	199	0.516	197	0.42	203	0.399	0.021	94.74%
HS117	0.824	0.394	0.064	0.632	41	0.653	47	0.549	37	0.588	0.039	93.37%
HS118	0.836	0.425	0.190	0.665	22	0.687	24	0.585	16	0.599	0.014	97.66%
HS119	0.823	0.328	0.297	0.624	45	0.648	51	0.557	30	0.59	0.033	94.41%
HS120	0.800	0.658	0.199	0.773	1	0.811	1	0.659	2	0.571	0.088	84.59%
HS121	0.891	0.230	0.096	0.596	71	0.635	58	0.522	64	0.495	0.027	94.55%
HS122	0.925	0.039	0.079	0.522	149	0.570	125	0.462	134	0.395	0.067	83.04%
HS123	0.711	0.304	0.126	0.540	129	0.568	130	0.47	124	0.425	0.045	89.41%
HS124	0.441	0.281	0.088	0.386	314	0.404	317	0.329	314	0.363	0.034	90.63%
HS125	0.639	0.165	0.096	0.432	267	0.459	265	0.378	255	0.371	0.007	98.11%
HS126	0.700	0.122	0.066	0.444	247	0.480	239	0.386	245	0.342	0.044	87.13%
HS127	0.690	0.114	0.003	0.429	273	0.463	257	0.369	267	0.324	0.045	86.11%
HS128	0.495	0.532	0.127	0.550	114	0.583	111	0.456	144	0.438	0.018	95.89%
HS129	0.593	0.265	0.062	0.457	232	0.487	226	0.39	237	0.364	0.026	92.86%
HS130	0.487	0.220	0.000	0.376	322	0.406	316	0.314	331	0.27	0.044	83.70%
HS131	0.476	0.447	0.183	0.502	174	0.530	179	0.423	188	0.406	0.017	95.81%
HS132	0.532	0.290	0.200	0.454	238	0.486	229	0.391	236	0.353	0.038	89.24%
HS133	0.533	0.600	0.236	0.607	61	0.629	67	0.516	69	0.541	0.025	95.38%
HS134	0.640	0.360	0.096	0.531	136	0.563	138	0.454	146	0.449	0.005	98.89%
HS135	0.678	0.202	0.306	0.496	182	0.528	180	0.441	160	0.413	0.028	93.22%

HS136	0.677	0.294	0.176	0.523	147	0.550	148	0.457	141	0.482	0.025	94.81%
HS137	0.426	0.373	0.133	0.434	264	0.462	261	0.363	276	0.333	0.030	90.99%
HS138	0.669	0.529	0.103	0.636	40	0.675	31	0.533	51	0.457	0.076	83.37%
HS139	0.436	0.137	0.043	0.310	414	0.331	413	0.264	411	0.262	0.002	99.24%
HS140	0.556	0.393	0.126	0.512	162	0.546	154	0.431	178	0.386	0.045	88.34%
HS141	0.490	0.557	0.174	0.560	103	0.591	105	0.47	124	0.447	0.023	94.85%
HS142	0.725	0.099	0.153	0.457	233	0.483	232	0.402	224	0.306	0.096	68.63%
HS143	0.715	0.290	0.135	0.541	128	0.577	120	0.468	128	0.441	0.027	93.88%
HS144	0.446	0.183	0.141	0.348	356	0.373	349	0.301	351	0.328	0.027	91.77%
HS145	0.769	0.192	0.193	0.529	140	0.568	126	0.464	132	0.403	0.061	84.86%
HS146	0.520	0.203	0.142	0.398	304	0.426	296	0.344	301	0.321	0.023	92.83%
HS147	0.416	0.314	0.354	0.425	276	0.452	272	0.368	269	0.362	0.006	98.34%
HS148	0.637	0.276	0.025	0.482	205	0.517	195	0.41	214	0.402	0.008	98.01%
HS149	0.607	0.326	0.166	0.507	169	0.541	159	0.435	169	0.406	0.029	92.86%
HS150	0.632	0.216	0.292	0.487	192	0.533	171	0.423	188	0.338	0.085	74.85%
HS151	0.438	0.444	0.253	0.484	201	0.507	211	0.414	209	0.425	0.011	97.41%
HS152	0.436	0.142	0.321	0.342	365	0.363	362	0.307	343	0.319	0.012	96.24%
HS153	0.223	0.276	0.239	0.290	434	0.303	440	0.246	436	0.272	0.026	90.44%
HS154	0.460	0.333	0.231	0.439	256	0.465	254	0.378	255	0.374	0.004	98.93%
HS155	0.543	0.245	0.291	0.444	248	0.472	243	0.392	235	0.393	0.001	99.75%
HS156	0.285	0.360	0.357	0.382	320	0.401	321	0.324	321	0.331	0.007	97.89%
HS157	0.459	0.354	0.256	0.456	235	0.483	235	0.39	237	0.387	0.003	99.22%
HS158	0.537	0.264	0.129	0.434	263	0.463	259	0.373	264	0.376	0.003	99.20%
HS159	0.244	0.339	0.124	0.321	399	0.337	400	0.263	415	0.282	0.019	93.26%
HS160	0.301	0.299	0.096	0.327	388	0.344	387	0.271	399	0.29	0.019	93.45%
HS161	0.303	0.316	0.187	0.345	361	0.360	363	0.291	368	0.309	0.018	94.17%
HS162	0.269	0.502	0.274	0.432	268	0.449	278	0.359	280	0.385	0.026	93.25%
HS163	0.909	0.350	0.112	0.667	20	0.710	13	0.579	21	0.491	0.088	82.08%

HS164	0.767	0.381	0.172	0.609	60	0.639	55	0.533	51	0.547	0.014	97.44%
HS165	0.770	0.310	0.101	0.573	89	0.601	92	0.497	92	0.473	0.024	94.93%
HS166	0.756	0.445	0.160	0.637	35	0.666	37	0.551	35	0.516	0.035	93.22%
HS167	0.727	0.230	0.098	0.508	167	0.534	169	0.445	157	0.464	0.019	95.91%
HS168	0.657	0.277	0.187	0.504	173	0.524	188	0.443	158	0.463	0.020	95.68%
HS169	0.640	0.316	0.117	0.514	158	0.553	147	0.44	161	0.381	0.059	84.51%
HS170	0.578	0.525	0.191	0.593	74	0.623	72	0.502	82	0.473	0.029	93.87%
HS171	0.577	0.372	0.046	0.502	175	0.533	173	0.422	196	0.388	0.034	91.24%
HS172	0.519	0.322	0.138	0.455	236	0.482	236	0.388	241	0.386	0.002	99.48%
HS173	0.685	0.230	0.078	0.487	196	0.514	201	0.422	196	0.427	0.005	98.83%
HS174	0.669	0.377	0.028	0.544	126	0.574	123	0.464	132	0.447	0.017	96.20%
HS175	0.840	0.143	0.080	0.524	145	0.557	145	0.462	134	0.413	0.049	88.14%
HS176	0.540	0.427	0.146	0.520	152	0.546	153	0.439	162	0.404	0.035	91.34%
HS177	0.723	0.385	0.096	0.584	78	0.608	84	0.503	80	0.505	0.002	99.60%
HS178	0.396	0.689	0.154	0.572	90	0.591	103	0.473	118	0.478	0.005	98.95%
HS179	0.413	0.396	0.066	0.436	261	0.461	262	0.356	286	0.292	0.064	78.08%
HS180	0.412	0.873	0.128	0.668	19	0.687	23	0.549	37	0.559	0.010	98.21%
HS181	0.788	0.417	0.220	0.642	32	0.669	35	0.563	27	0.558	0.005	99.10%
HS182	0.546	0.613	0.235	0.617	52	0.633	61	0.527	58	0.542	0.015	97.23%
HS183	0.648	0.502	0.501	0.648	31	0.673	34	0.571	25	0.583	0.012	97.94%
HS184	0.804	0.327	0.266	0.612	58	0.636	57	0.543	43	0.555	0.012	97.84%
HS185	0.769	0.274	0.197	0.562	102	0.591	104	0.496	94	0.482	0.014	97.10%
HS186	0.794	0.358	0.067	0.603	66	0.630	66	0.522	64	0.509	0.013	97.45%
HS187	0.843	0.288	0.261	0.617	53	0.650	50	0.546	41	0.51	0.036	92.94%
HS188	0.673	0.178	0.196	0.470	217	0.499	215	0.414	209	0.39	0.024	93.85%
HS189	0.794	0.076	0.122	0.471	216	0.504	212	0.421	199	0.398	0.023	94.22%
HS190	0.717	0.047	0.224	0.433	265	0.467	248	0.388	241	0.352	0.036	89.77%
HS191	0.821	0.183	0.366	0.565	98	0.601	91	0.51	75	0.5	0.010	98.00%

HS192	0.880	0.406	1.000	0.769	2	0.804	2	0.715	1	0.771	0.056	92.74%
HS193	0.323	0.317	0.217	0.361	340	0.381	341	0.305	347	0.293	0.012	95.90%
HS194	0.601	0.273	0.239	0.484	200	0.514	198	0.423	188	0.379	0.044	88.39%
HS195	0.356	0.158	0.206	0.298	428	0.317	425	0.258	424	0.269	0.011	95.91%
HS196	0.269	0.491	0.248	0.428	275	0.451	276	0.351	291	0.33	0.021	93.64%
HS197	0.447	0.142	0.235	0.344	363	0.372	351	0.299	354	0.277	0.022	92.06%
HS198	0.421	0.205	0.253	0.361	341	0.385	337	0.314	331	0.309	0.005	98.38%
HS199	0.428	0.045	0.231	0.282	443	0.306	434	0.253	431	0.254	0.001	99.61%
HS200	0.518	0.369	0.606	0.532	135	0.565	134	0.473	118	0.426	0.047	88.97%
HS201	0.595	0.311	0.931	0.577	86	0.612	82	0.534	50	0.518	0.016	96.91%
HS202	0.620	0.061	0.252	0.390	310	0.422	303	0.352	289	0.325	0.027	91.69%
HS203	0.454	0.142	0.119	0.331	382	0.358	365	0.285	378	0.252	0.033	86.90%
HS204	0.465	0.175	0.088	0.349	354	0.375	347	0.299	354	0.276	0.023	91.67%
HS205	0.392	0.231	0.268	0.358	347	0.378	343	0.312	334	0.316	0.004	98.73%
HS206	0.354	0.119	0.088	0.264	464	0.280	462	0.226	464	0.257	0.031	87.94%
HS207	0.510	0.239	0.276	0.422	280	0.444	283	0.372	265	0.38	0.008	97.89%
HS208	0.440	0.243	0.137	0.372	329	0.390	333	0.32	324	0.343	0.023	93.29%
HS209	0.380	0.359	0.130	0.401	302	0.422	301	0.336	308	0.343	0.007	97.96%
HS210	0.351	0.409	0.397	0.442	250	0.467	249	0.38	251	0.381	0.001	99.74%
HS211	0.416	0.853	0.163	0.670	18	0.702	16	0.548	39	0.53	0.018	96.60%
HS212	0.357	0.355	0.091	0.384	318	0.402	320	0.318	327	0.341	0.023	93.26%
HS213	0.486	0.616	0.125	0.580	83	0.598	95	0.484	103	0.469	0.015	96.80%
HS214	0.526	0.440	0.112	0.514	160	0.536	166	0.433	172	0.425	0.008	98.12%
HS215	0.461	0.486	0.080	0.495	184	0.514	200	0.416	206	0.446	0.030	93.27%
HS216	0.512	0.490	0.024	0.519	154	0.540	160	0.433	172	0.433	0.000	100.00%
HS217	0.539	0.805	0.113	0.686	14	0.691	19	0.58	20	0.677	0.097	85.67%
HS218	0.620	0.461	0.036	0.554	110	0.564	136	0.475	115	0.502	0.027	94.62%
HS219	0.606	0.467	0.023	0.558	106	0.585	110	0.468	128	0.443	0.025	94.36%

HS220	0.469	1.000	0.193	0.756	3	0.761	4	0.634	5	0.71	0.076	89.30%
HS221	0.545	0.760	0.083	0.672	17	0.691	20	0.561	28	0.548	0.013	97.63%
HS222	0.409	0.611	0.057	0.526	143	0.538	163	0.436	167	0.49	0.054	88.98%
HS223	0.765	0.364	0.296	0.624	46	0.658	44	0.543	43	0.48	0.063	86.88%
HS224	0.917	0.432	0.370	0.734	4	0.766	3	0.652	3	0.623	0.029	95.35%
HS225	0.814	0.495	0.244	0.700	9	0.728	9	0.609	8	0.589	0.020	96.60%
HS226	0.740	0.344	0.198	0.583	80	0.609	83	0.51	75	0.5	0.010	98.00%
HS227	0.687	0.339	0.157	0.546	124	0.567	131	0.477	113	0.486	0.009	98.15%
HS228	0.918	0.100	0.283	0.571	91	0.622	74	0.511	74	0.426	0.085	80.05%
HS229	0.747	0.176	0.106	0.491	190	0.514	199	0.435	169	0.445	0.010	97.75%
HS230	0.831	0.287	0.152	0.600	69	0.632	65	0.524	61	0.493	0.031	93.71%
HS231	0.805	0.193	0.232	0.550	115	0.585	109	0.487	101	0.442	0.045	89.82%
HS232	0.734	0.260	0.256	0.543	127	0.568	127	0.483	104	0.463	0.020	95.68%
HS233	0.794	0.148	0.283	0.521	151	0.548	150	0.472	121	0.484	0.012	97.52%
HS234	0.817	0.105	0.243	0.511	164	0.544	156	0.46	139	0.441	0.019	95.69%
HS235	0.818	0.276	0.707	0.654	29	0.702	15	0.593	12	0.532	0.061	88.53%
HS236	0.586	0.161	0.091	0.404	296	0.433	292	0.351	291	0.337	0.014	95.85%
HS237	0.688	0.276	0.218	0.530	138	0.564	135	0.462	134	0.415	0.047	88.67%
HS238	0.758	0.512	0.305	0.689	13	0.719	10	0.598	10	0.597	0.001	99.83%
HS239	0.738	0.238	0.291	0.537	131	0.557	144	0.481	107	0.495	0.014	97.17%
HS240	0.832	0.226	0.161	0.567	95	0.595	99	0.502	82	0.506	0.004	99.21%
HS241	0.736	0.142	0.172	0.483	204	0.518	193	0.426	186	0.382	0.044	88.48%
HS242	0.660	0.239	0.245	0.499	177	0.537	164	0.438	163	0.352	0.086	75.57%
HS243	0.451	0.001	0.044	0.248	476	0.270	472	0.219	467	0.25	0.031	87.60%
HS244	0.696	0.173	0.305	0.494	185	0.534	168	0.438	163	0.367	0.071	80.65%
HS245	0.673	0.202	0.365	0.504	171	0.545	155	0.448	153	0.369	0.079	78.59%
HS246	0.390	0.112	0.205	0.292	432	0.314	428	0.257	426	0.253	0.004	98.42%
HS247	0.658	0.110	0.188	0.428	274	0.463	258	0.379	253	0.351	0.028	92.02%

HS248	0.404	0.261	0.513	0.411	289	0.438	289	0.365	272	0.352	0.013	96.31%
HS249	0.455	0.308	0.183	0.418	285	0.443	284	0.359	280	0.327	0.032	90.21%
HS250	0.661	0.208	0.248	0.486	197	0.524	187	0.427	185	0.356	0.071	80.06%
HS251	0.652	0.245	0.221	0.495	183	0.531	178	0.433	172	0.402	0.031	92.29%
HS252	0.324	0.224	0.196	0.316	408	0.337	401	0.267	407	0.246	0.021	91.46%
HS253	0.452	0.250	0.178	0.394	308	0.423	300	0.335	309	0.313	0.022	92.97%
HS254	0.557	0.208	0.310	0.437	260	0.465	253	0.387	243	0.374	0.013	96.52%
HS255	0.450	0.436	0.259	0.493	187	0.520	190	0.417	204	0.387	0.030	92.25%
HS256	0.529	0.374	0.256	0.497	178	0.525	183	0.43	180	0.371	0.059	84.10%
HS257	0.500	0.397	0.425	0.515	157	0.539	161	0.45	151	0.423	0.027	93.62%
HS258	0.548	0.007	0.223	0.321	400	0.346	382	0.293	363	0.317	0.024	92.43%
HS259	0.493	0.143	0.267	0.368	333	0.396	325	0.326	317	0.307	0.019	93.81%
HS260	0.467	0.038	0.206	0.296	431	0.320	422	0.264	411	0.262	0.002	99.24%
HS261	0.484	0.129	0.230	0.354	350	0.382	340	0.311	337	0.287	0.024	91.64%
HS262	0.587	0.228	0.282	0.459	227	0.492	221	0.405	220	0.381	0.024	93.70%
HS263	0.587	0.221	0.283	0.454	237	0.484	231	0.402	224	0.402	0.000	100.00%
HS264	0.397	0.419	0.354	0.463	224	0.483	233	0.399	227	0.4	0.001	99.75%
HS265	0.199	0.284	0.062	0.261	469	0.269	474	0.212	477	0.258	0.046	82.17%
HS266	0.322	0.241	0.221	0.319	404	0.333	407	0.276	390	0.339	0.063	81.42%
HS267	0.345	0.132	0.113	0.267	461	0.283	459	0.229	458	0.273	0.044	83.88%
HS268	0.311	0.268	0.242	0.331	381	0.348	381	0.284	379	0.315	0.031	90.16%
HS269	0.549	0.249	0.279	0.451	239	0.481	238	0.395	231	0.406	0.011	97.29%
HS270	0.655	0.222	0.203	0.485	198	0.520	192	0.423	188	0.393	0.030	92.37%
HS271	0.660	0.344	0.096	0.536	132	0.564	137	0.457	141	0.393	0.064	83.72%
HS272	0.710	0.344	0.188	0.566	97	0.593	101	0.494	96	0.49	0.004	99.18%
HS273	0.546	0.594	0.201	0.605	63	0.625	70	0.515	70	0.545	0.030	94.50%
HS274	0.703	0.317	0.062	0.547	121	0.586	107	0.462	134	0.391	0.071	81.84%
HS275	0.701	0.247	0.050	0.497	179	0.527	181	0.432	175	0.41	0.022	94.63%

HS276	0.476	0.648	0.283	0.605	64	0.616	80	0.514	72	0.541	0.027	95.01%
HS277	0.621	0.500	0.098	0.595	73	0.623	73	0.499	86	0.442	0.057	87.10%
HS278	0.653	0.517	0.122	0.617	54	0.642	53	0.525	60	0.516	0.009	98.26%
HS279	0.576	0.330	0.174	0.500	176	0.538	162	0.423	188	0.378	0.045	88.10%
HS280	0.204	0.540	0.150	0.404	297	0.417	309	0.326	317	0.332	0.006	98.19%
HS281	0.801	0.597	0.176	0.732	5	0.749	5	0.633	6	0.629	0.004	99.36%
HS282	0.607	0.774	0.216	0.725	6	0.742	8	0.615	7	0.653	0.038	94.18%
HS283	0.574	0.466	0.176	0.553	113	0.572	124	0.475	115	0.504	0.029	94.25%
HS284	0.794	0.446	0.245	0.658	24	0.678	29	0.581	19	0.651	0.070	89.25%
HS285	0.600	0.407	0.223	0.550	118	0.580	117	0.472	121	0.45	0.022	95.11%
HS286	0.896	0.411	0.187	0.701	8	0.744	7	0.607	9	0.545	0.062	88.62%
HS287	0.450	0.327	0.149	0.421	281	0.439	287	0.36	278	0.357	0.003	99.16%
HS288	0.383	0.311	0.120	0.375	326	0.393	327	0.318	327	0.327	0.009	97.25%
HS289	0.851	0.267	0.169	0.599	70	0.632	63	0.528	56	0.504	0.024	95.24%
HS290	0.863	0.277	0.192	0.612	59	0.639	54	0.541	47	0.53	0.011	97.92%
HS291	0.621	0.428	0.181	0.560	105	0.582	114	0.483	104	0.494	0.011	97.77%
HS292	0.795	0.117	0.125	0.494	186	0.531	177	0.438	163	0.39	0.048	87.69%
HS293	0.929	0.279	0.309	0.654	28	0.684	26	0.589	13	0.62	0.031	95.00%
HS294	0.848	0.284	0.176	0.601	67	0.619	77	0.535	49	0.591	0.056	90.52%
HS295	0.737	0.465	0.186	0.637	37	0.662	39	0.553	34	0.548	0.005	99.09%
HS296	0.736	0.334	0.128	0.571	93	0.604	88	0.494	96	0.471	0.023	95.12%
HS297	0.874	0.183	0.118	0.567	96	0.605	86	0.499	86	0.497	0.002	99.60%
HS298	0.832	0.350	0.195	0.637	38	0.673	33	0.555	33	0.514	0.041	92.02%
HS299	0.585	0.159	0.326	0.432	266	0.465	252	0.384	248	0.356	0.028	92.13%
HS300	0.598	0.435	0.275	0.568	94	0.599	93	0.489	100	0.471	0.018	96.18%
HS301	0.263	0.269	0.214	0.305	422	0.319	424	0.258	424	0.272	0.014	94.85%
HS302	0.391	0.306	0.388	0.409	292	0.432	293	0.358	283	0.34	0.018	94.71%
HS303	0.250	0.322	0.206	0.324	394	0.340	394	0.271	399	0.269	0.002	99.26%

HS304	0.288	0.212	0.261	0.298	426	0.317	426	0.255	430	0.248	0.007	97.18%
HS305	0.625	0.086	0.219	0.410	291	0.452	273	0.359	280	0.284	0.075	73.59%
HS306	0.449	0.177	0.209	0.358	346	0.384	338	0.31	338	0.275	0.035	87.27%
HS307	0.297	0.253	0.295	0.322	398	0.339	397	0.28	385	0.287	0.007	97.56%
HS308	0.387	0.100	0.175	0.283	441	0.306	436	0.246	436	0.233	0.013	94.42%
HS309	0.394	0.086	0.184	0.280	446	0.305	439	0.245	439	0.234	0.011	95.30%
HS310	0.382	0.043	0.142	0.248	478	0.267	475	0.217	472	0.22	0.003	98.64%
HS311	0.271	0.322	0.561	0.375	324	0.392	328	0.333	311	0.321	0.012	96.26%
HS312	0.430	0.152	0.004	0.311	413	0.331	411	0.262	416	0.242	0.020	91.74%
HS313	0.388	0.167	0.119	0.308	417	0.327	417	0.264	411	0.265	0.001	99.62%
HS314	0.347	0.084	0.157	0.252	474	0.272	469	0.218	469	0.22	0.002	99.09%
HS315	0.518	0.286	0.173	0.442	251	0.469	247	0.379	253	0.361	0.018	95.01%
HS316	0.372	0.316	0.122	0.371	330	0.385	335	0.314	331	0.34	0.026	92.35%
HS317	0.521	0.285	0.323	0.457	229	0.481	237	0.402	224	0.409	0.007	98.29%
HS318	0.451	0.331	0.144	0.429	272	0.455	269	0.361	277	0.334	0.027	91.92%
HS319	0.191	0.261	0.110	0.247	480	0.251	480	0.206	481	0.283	0.077	72.79%
HS320	0.232	0.278	0.100	0.277	451	0.285	456	0.231	457	0.288	0.057	80.21%
HS321	0.521	0.275	0.101	0.429	271	0.451	275	0.366	271	0.365	0.001	99.73%
HS322	0.401	0.356	0.172	0.416	287	0.435	290	0.351	291	0.345	0.006	98.26%
HS323	0.000	0.448	0.117	0.249	475	0.251	482	0.189	489	0.238	0.049	79.41%
HS324	0.080	0.509	0.113	0.320	402	0.324	420	0.25	433	0.272	0.022	91.91%
HS325	0.537	0.274	0.129	0.437	258	0.457	266	0.377	258	0.387	0.010	97.42%
HS326	0.323	0.267	0.122	0.325	391	0.341	391	0.273	395	0.289	0.016	94.46%
HS327	0.394	0.412	0.161	0.439	255	0.463	260	0.368	269	0.354	0.014	96.05%
HS328	0.417	0.506	0.129	0.489	191	0.508	208	0.41	214	0.435	0.025	94.25%
HS329	0.417	0.294	0.107	0.386	315	0.407	315	0.325	319	0.297	0.028	90.57%
HS330	0.342	0.649	0.161	0.528	142	0.543	157	0.434	171	0.427	0.007	98.36%
HS331	0.455	0.376	0.131	0.447	244	0.469	246	0.378	255	0.366	0.012	96.72%

HS332	0.367	0.406	0.214	0.424	277	0.442	286	0.36	278	0.373	0.013	96.51%
HS333	0.642	0.215	0.053	0.457	230	0.487	228	0.393	233	0.372	0.021	94.35%
HS334	0.364	0.246	0.139	0.337	374	0.353	378	0.287	373	0.29	0.003	98.97%
HS335	0.304	0.383	0.105	0.365	337	0.373	350	0.306	344	0.327	0.021	93.58%
HS336	0.783	0.146	0.218	0.508	168	0.532	175	0.457	141	0.478	0.021	95.61%
HS337	0.414	0.194	0.208	0.340	370	0.354	374	0.3	353	0.312	0.012	96.15%
HS338	0.212	0.402	0.118	0.332	380	0.343	389	0.272	397	0.298	0.026	91.28%
HS339	0.169	0.367	0.093	0.287	438	0.292	449	0.234	451	0.266	0.032	87.97%
HS340	0.351	0.634	0.092	0.517	155	0.536	167	0.423	188	0.445	0.022	95.06%
HS341	0.525	0.199	0.170	0.392	309	0.410	311	0.348	297	0.385	0.037	90.39%
HS342	0.491	0.011	0.183	0.281	445	0.294	446	0.262	416	0.353	0.091	74.22%
HS343	0.328	0.394	0.130	0.385	316	0.399	322	0.325	319	0.344	0.019	94.48%
HS344	0.381	0.194	0.126	0.315	410	0.331	414	0.272	397	0.29	0.018	93.79%
HS345	0.331	0.484	0.265	0.449	242	0.463	256	0.38	251	0.419	0.039	90.69%
HS346	0.321	0.258	0.110	0.317	407	0.333	410	0.266	409	0.255	0.011	95.69%
HS347	0.273	0.150	0.157	0.247	479	0.260	479	0.209	479	0.219	0.010	95.43%
HS348	0.613	0.214	0.226	0.460	226	0.489	224	0.403	223	0.385	0.018	95.32%
HS349	0.300	0.296	0.260	0.341	367	0.355	370	0.293	363	0.304	0.011	96.38%
HS350	0.267	0.329	0.241	0.339	371	0.350	380	0.287	373	0.306	0.019	93.79%
HS351	0.424	0.403	0.150	0.441	253	0.456	268	0.377	258	0.427	0.050	88.29%
HS352	0.293	0.302	0.283	0.345	360	0.358	366	0.295	362	0.322	0.027	91.61%
HS353	0.350	0.294	0.359	0.375	323	0.391	330	0.33	313	0.342	0.012	96.49%
HS354	0.416	0.296	0.165	0.390	311	0.408	314	0.334	310	0.355	0.021	94.08%
HS355	0.230	0.259	0.213	0.283	442	0.292	450	0.239	445	0.283	0.044	84.45%
HS356	0.270	0.326	0.274	0.344	362	0.357	367	0.292	366	0.326	0.034	89.57%
HS357	0.417	0.289	0.351	0.405	294	0.422	304	0.358	283	0.403	0.045	88.83%
HS358	0.301	0.333	0.252	0.358	345	0.371	352	0.306	344	0.348	0.042	87.93%
HS359	0.287	0.354	0.252	0.359	344	0.365	359	0.308	340	0.381	0.073	80.84%

HS360	0.239	0.202	0.183	0.258	471	0.271	471	0.217	472	0.23	0.013	94.35%
HS361	0.203	0.311	0.090	0.279	449	0.291	451	0.228	460	0.282	0.054	80.85%
HS362	0.494	0.344	0.269	0.449	243	0.466	250	0.404	222	0.612	0.208	66.01%
HS363	0.091	0.326	0.165	0.239	483	0.244	488	0.192	487	0.255	0.063	75.29%
HS364	0.236	0.257	0.273	0.291	433	0.305	438	0.25	433	0.278	0.028	89.93%
HS365	0.198	0.298	0.185	0.283	440	0.293	447	0.235	450	0.265	0.030	88.68%
HS366	0.438	0.153	0.182	0.331	383	0.345	385	0.292	366	0.331	0.039	88.22%
HS367	0.212	0.296	0.134	0.280	448	0.287	454	0.233	453	0.283	0.050	82.33%
HS368	0.478	0.223	0.180	0.389	312	0.410	310	0.337	304	0.356	0.019	94.66%
HS369	0.405	0.327	0.227	0.407	293	0.421	306	0.349	295	0.383	0.034	91.12%
HS370	0.254	0.309	0.246	0.323	395	0.333	408	0.274	394	0.309	0.035	88.67%
HS371	0.331	0.179	0.260	0.299	425	0.314	429	0.262	416	0.298	0.036	87.92%
HS372	0.353	0.347	0.168	0.387	313	0.409	312	0.324	321	0.295	0.029	90.17%
HS373	0.217	0.250	0.193	0.269	459	0.282	460	0.226	464	0.266	0.040	84.96%
HS374	0.366	0.282	0.216	0.366	335	0.385	336	0.312	334	0.328	0.016	95.12%
HS375	0.284	0.286	0.181	0.320	401	0.336	403	0.27	402	0.277	0.007	97.47%
HS376	0.341	0.259	0.282	0.348	355	0.367	357	0.301	351	0.322	0.021	93.48%
HS377	0.243	0.223	0.116	0.261	467	0.274	466	0.217	472	0.25	0.033	86.80%
HS378	0.369	0.267	0.153	0.356	348	0.380	342	0.299	354	0.286	0.013	95.45%
HS379	0.357	0.303	0.164	0.360	342	0.371	353	0.308	340	0.364	0.056	84.62%
HS380	0.385	0.216	0.233	0.345	359	0.364	360	0.298	357	0.306	0.008	97.39%
HS381	0.275	0.130	0.135	0.234	486	0.249	484	0.199	483	0.222	0.023	89.64%
HS382	0.271	0.210	0.129	0.270	458	0.283	458	0.227	462	0.256	0.029	88.67%
HS383	0.153	0.188	0.149	0.201	497	0.210	498	0.166	498	0.203	0.037	81.77%
HS384	0.274	0.044	0.099	0.183	501	0.194	501	0.16	500	0.229	0.069	69.87%
HS385	0.250	0.375	0.151	0.341	368	0.353	376	0.284	379	0.319	0.035	89.03%
HS386	0.551	0.168	0.280	0.410	290	0.433	291	0.364	273	0.356	0.008	97.75%
HS387	0.349	0.488	0.230	0.456	234	0.472	244	0.386	245	0.403	0.017	95.78%

HS388	0.311	0.373	0.081	0.366	336	0.383	339	0.302	350	0.305	0.003	99.02%
HS389	0.320	0.279	0.240	0.341	369	0.355	369	0.293	363	0.309	0.016	94.82%
HS390	0.538	0.224	0.211	0.421	282	0.439	288	0.37	266	0.362	0.008	97.79%
HS391	0.273	0.292	0.256	0.324	393	0.338	398	0.277	389	0.312	0.035	88.78%
HS392	0.515	0.295	0.186	0.441	254	0.460	263	0.383	249	0.399	0.016	95.99%
HS393	0.548	0.252	0.194	0.441	252	0.464	255	0.383	249	0.373	0.010	97.32%
HS394	0.425	0.224	0.162	0.359	343	0.376	345	0.31	338	0.323	0.013	95.98%
HS395	0.613	0.609	0.001	0.630	43	0.659	43	0.523	63	0.478	0.045	90.59%
HS396	0.281	0.255	0.154	0.300	424	0.312	430	0.253	431	0.272	0.019	93.01%
HS397	0.450	0.344	0.215	0.431	269	0.446	282	0.376	260	0.428	0.052	87.85%
HS398	0.530	0.101	0.140	0.350	353	0.376	346	0.308	340	0.292	0.016	94.52%
HS399	0.401	0.069	0.142	0.265	462	0.280	461	0.236	449	0.292	0.056	80.82%
HS400	0.307	0.353	0.033	0.348	357	0.364	361	0.286	376	0.314	0.028	91.08%
HS401	0.384	0.272	0.079	0.350	352	0.367	358	0.297	360	0.32	0.023	92.81%
HS402	0.559	0.307	0.211	0.473	214	0.497	219	0.412	212	0.392	0.020	94.90%
HS403	0.504	0.212	0.230	0.400	303	0.423	298	0.352	289	0.376	0.024	93.62%
HS404	0.605	0.342	0.214	0.514	159	0.532	176	0.447	155	0.474	0.027	94.30%
HS405	0.432	0.301	0.219	0.403	298	0.418	308	0.351	291	0.382	0.031	91.88%
HS406	0.376	0.279	0.293	0.378	321	0.396	323	0.327	316	0.342	0.015	95.61%
HS407	0.600	0.000	0.144	0.339	372	0.369	355	0.303	349	0.298	0.005	98.32%
HS408	0.397	0.065	0.141	0.261	466	0.277	465	0.232	456	0.288	0.056	80.56%
HS409	0.304	0.044	0.177	0.212	492	0.228	491	0.186	491	0.212	0.026	87.74%
HS410	0.351	0.111	0.282	0.273	457	0.287	453	0.249	435	0.322	0.073	77.33%
HS411	0.147	0.096	0.169	0.158	502	0.168	502	0.131	502	0.145	0.014	90.34%
HS412	0.331	0.196	0.179	0.297	429	0.310	431	0.257	426	0.305	0.048	84.26%
HS413	0.332	0.095	0.359	0.276	454	0.297	443	0.245	439	0.214	0.031	85.51%
HS414	0.345	0.052	0.113	0.230	488	0.248	486	0.199	483	0.216	0.017	92.13%
HS415	0.269	0.176	0.210	0.261	468	0.274	467	0.225	466	0.26	0.035	86.54%

HS416	0.259	0.154	0.161	0.239	482	0.250	483	0.204	482	0.252	0.048	80.95%
HS417	0.451	0.133	0.117	0.322	396	0.341	393	0.281	382	0.31	0.029	90.65%
HS418	0.450	0.123	0.191	0.329	387	0.355	373	0.287	373	0.274	0.013	95.26%
HS419	0.391	0.152	0.171	0.309	416	0.331	412	0.268	405	0.262	0.006	97.71%
HS420	0.409	0.080	0.124	0.279	450	0.301	441	0.241	442	0.24	0.001	99.58%
HS421	0.295	0.151	0.147	0.255	472	0.269	473	0.219	467	0.256	0.037	85.55%
HS422	0.488	0.073	0.152	0.319	405	0.344	386	0.28	385	0.274	0.006	97.81%
HS423	0.271	0.116	0.255	0.238	484	0.251	481	0.209	479	0.244	0.035	85.66%
HS424	0.373	0.287	0.217	0.370	331	0.392	329	0.318	327	0.318	0.000	100.00%
HS425	0.384	0.079	0.125	0.265	463	0.283	457	0.229	458	0.243	0.014	94.24%
HS426	0.306	0.239	0.166	0.307	421	0.325	419	0.26	421	0.266	0.006	97.74%
HS427	0.226	0.211	0.207	0.254	473	0.265	476	0.217	472	0.248	0.031	87.50%
HS428	0.494	0.074	0.200	0.325	392	0.346	383	0.29	370	0.314	0.024	92.36%
HS429	0.325	0.231	0.234	0.319	406	0.335	405	0.276	390	0.286	0.010	96.50%
HS430	0.329	0.212	0.145	0.307	420	0.329	415	0.257	426	0.238	0.019	92.02%
HS431	0.415	0.181	0.162	0.334	377	0.355	372	0.288	371	0.279	0.009	96.77%
HS432	0.291	0.342	0.202	0.355	349	0.374	348	0.298	357	0.289	0.009	96.89%
HS433	0.454	0.166	0.301	0.362	338	0.386	334	0.321	323	0.33	0.009	97.27%
HS434	0.150	0.456	0.209	0.341	366	0.353	379	0.276	390	0.303	0.027	91.09%
HS435	0.373	0.570	0.218	0.510	165	0.525	184	0.426	186	0.449	0.023	94.88%
HS436	0.581	0.282	0.186	0.476	211	0.508	206	0.409	216	0.373	0.036	90.35%
HS437	0.418	0.428	0.234	0.467	221	0.491	222	0.395	231	0.375	0.020	94.67%
HS438	0.208	0.437	0.220	0.362	339	0.376	344	0.298	357	0.32	0.022	93.13%
HS439	0.277	0.545	0.109	0.438	257	0.457	267	0.356	286	0.376	0.020	94.68%
HS440	0.242	0.653	0.067	0.470	218	0.487	227	0.375	262	0.373	0.002	99.46%
HS441	0.266	0.468	0.158	0.403	300	0.422	302	0.328	315	0.331	0.003	99.09%
HS442	0.200	0.418	0.075	0.329	386	0.340	395	0.266	409	0.303	0.037	87.79%
HS443	0.109	0.523	0.078	0.336	375	0.343	390	0.264	411	0.311	0.047	84.89%

HS444	0.111	0.486	0.194	0.330	384	0.336	404	0.267	407	0.319	0.052	83.70%
HS445	0.179	0.217	0.201	0.235	485	0.245	487	0.197	485	0.225	0.028	87.56%
HS446	0.203	0.599	0.153	0.435	262	0.452	274	0.348	297	0.338	0.010	97.04%
HS447	0.369	0.363	0.175	0.402	301	0.420	307	0.339	303	0.357	0.018	94.96%
HS448	0.560	0.150	0.166	0.394	306	0.424	297	0.345	299	0.329	0.016	95.14%
HS449	0.317	0.205	0.199	0.302	423	0.320	423	0.257	426	0.263	0.006	97.72%
HS450	0.286	0.343	0.207	0.354	351	0.370	354	0.296	361	0.322	0.026	91.93%
HS451	0.230	0.488	0.247	0.405	295	0.423	299	0.332	312	0.317	0.015	95.27%
HS452	0.451	0.291	0.227	0.420	283	0.450	277	0.357	285	0.323	0.034	89.47%
HS453	0.540	0.257	0.309	0.457	231	0.490	223	0.398	229	0.351	0.047	86.61%
HS454	0.224	0.271	0.160	0.280	447	0.293	448	0.233	453	0.26	0.027	89.62%
HS455	0.600	0.338	0.245	0.522	150	0.559	140	0.448	153	0.4	0.048	88.00%
HS456	0.562	0.232	0.241	0.444	249	0.471	245	0.389	239	0.372	0.017	95.43%
HS457	0.240	0.121	0.116	0.207	495	0.215	497	0.176	496	0.226	0.050	77.88%
HS458	0.505	0.297	0.452	0.469	219	0.492	220	0.417	204	0.436	0.019	95.64%
HS459	0.384	0.201	0.158	0.326	390	0.341	392	0.281	382	0.308	0.027	91.23%
HS460	0.198	0.310	0.175	0.287	439	0.296	444	0.238	446	0.269	0.031	88.48%
HS461	0.323	0.352	0.226	0.382	319	0.403	319	0.32	324	0.312	0.008	97.44%
HS462	0.310	0.170	0.178	0.277	453	0.290	452	0.237	448	0.252	0.015	94.05%
HS463	0.446	0.285	0.309	0.418	286	0.442	285	0.364	273	0.381	0.017	95.54%
HS464	0.522	0.337	0.250	0.477	210	0.504	214	0.412	212	0.406	0.006	98.52%
HS465	0.334	0.195	0.265	0.309	415	0.324	421	0.27	402	0.294	0.024	91.84%
HS466	0.315	0.331	0.248	0.370	332	0.390	331	0.312	334	0.3	0.012	96.00%
HS467	0.370	0.308	0.303	0.394	307	0.421	305	0.337	304	0.336	0.001	99.70%
HS468	0.127	0.332	0.187	0.264	465	0.271	470	0.215	476	0.248	0.033	86.69%
HS469	0.202	0.251	0.242	0.269	460	0.280	463	0.227	462	0.23	0.003	98.70%
HS470	0.125	0.409	0.161	0.298	427	0.307	433	0.24	443	0.25	0.010	96.00%
HS471	0.185	0.206	0.158	0.227	489	0.234	490	0.189	489	0.229	0.040	82.53%

HS472	0.243	0.264	0.151	0.289	435	0.306	435	0.238	446	0.215	0.023	89.30%
HS473	0.159	0.218	0.146	0.218	491	0.228	493	0.18	492	0.217	0.037	82.95%
HS474	0.186	0.292	0.191	0.275	455	0.286	455	0.228	460	0.233	0.005	97.85%
HS475	0.159	0.416	0.175	0.322	397	0.336	402	0.26	421	0.257	0.003	98.83%
HS476	0.065	0.416	0.188	0.273	456	0.280	464	0.218	469	0.256	0.038	85.16%
HS477	0.327	0.238	0.159	0.316	409	0.334	406	0.269	404	0.248	0.021	91.53%
HS478	0.381	0.383	0.200	0.424	278	0.447	281	0.356	286	0.327	0.029	91.13%
HS479	0.392	0.425	0.188	0.446	245	0.466	251	0.376	260	0.34	0.036	89.41%
HS480	0.319	0.308	0.155	0.348	358	0.368	356	0.291	368	0.275	0.016	94.18%
HS481	0.347	0.250	0.133	0.333	379	0.353	377	0.279	387	0.252	0.027	89.29%
HS482	0.384	0.376	0.134	0.418	284	0.448	279	0.345	299	0.279	0.066	76.34%
HS483	0.297	0.076	0.067	0.211	493	0.228	492	0.179	493	0.208	0.029	86.06%
HS484	0.349	0.206	0.242	0.320	403	0.338	399	0.279	387	0.295	0.016	94.58%
HS485	0.382	0.199	0.215	0.334	378	0.357	368	0.288	371	0.282	0.006	97.87%
HS486	0.378	0.158	0.204	0.308	418	0.326	418	0.268	405	0.271	0.003	98.89%
HS487	0.418	0.148	0.118	0.315	411	0.340	396	0.271	399	0.267	0.004	98.50%
HS488	0.269	0.231	0.172	0.288	437	0.305	437	0.24	443	0.22	0.020	90.91%
HS489	0.271	0.109	0.151	0.222	490	0.237	489	0.191	488	0.227	0.036	84.14%
HS490	0.387	0.167	0.105	0.307	419	0.328	416	0.262	416	0.249	0.013	94.78%
HS491	0.247	0.065	0.104	0.188	500	0.205	500	0.156	501	0.135	0.021	84.44%
HS492	0.225	0.179	0.121	0.233	487	0.248	485	0.193	486	0.201	0.008	96.02%
HS493	0.332	0.086	0.197	0.248	477	0.262	478	0.218	469	0.236	0.018	92.37%
HS494	0.303	0.195	0.174	0.282	444	0.294	445	0.243	441	0.259	0.016	93.82%
HS495	0.274	0.064	0.165	0.202	496	0.215	496	0.178	494	0.225	0.047	79.11%
HS496	0.306	0.108	0.325	0.260	470	0.274	468	0.233	453	0.267	0.034	87.27%
HS497	0.284	0.139	0.159	0.247	481	0.264	477	0.21	478	0.23	0.020	91.30%
HS498	0.268	0.088	0.128	0.208	494	0.222	494	0.178	494	0.206	0.028	86.41%
HS499	0.330	0.022	0.041	0.201	498	0.217	495	0.17	497	0.177	0.007	96.05%

HS500	0.386	0.129	0.202	0.297	430	0.314	427	0.26	421	0.265	0.005	98.11%
HS501	0.237	0.089	0.132	0.195	499	0.209	499	0.165	499	0.188	0.023	87.77%
HS502	0.211	0.322	0.432	0.330	385	0.346	384	0.286	376	0.303	0.017	94.39%
Av				0.443		0.467		0.381				92.52%
CV				28.84%		29.18%		29.84%				
