# Supplementary material for: Screening and Comprehensive Evaluation of Drought Resistance in Cotton Germplasm Resources at the Germination Stage
Source: Plants (Basel). 2025 Jul 15;14(14):2191. doi: 10.3390/plants14142191 (PMC12299762; doi:10.3390/plants14142191)
Supplement: Supplementary file 1 [file plants-14-02191-s001.zip › plants-3738055-supplementary/plants-3738055-supplementary/Supplement/Supplementary File S2.pdf]

Table S3. BLUP Predicting D values with actual error.

Code	BLUP Predicting D values	D-Value of REP1	Dvalue of REP2	Error with REP1	Error with REP2
HS1	0.497	0.503	0.496	0.006	0.001
HS2	0.344	0.527	0.276	0.183	0.068
HS3	0.484	0.433	0.515	0.051	0.031
HS4	0.453	0.371	0.584	0.082	0.131
HS5	0.42	0.385	0.504	0.035	0.084
HS6	0.228	0.271	0.306	0.043	0.078
HS7	0.408	0.247	0.586	0.161	0.178
HS8	0.315	0.234	0.494	0.081	0.179
HS9	0.578	0.521	0.577	0.057	0.001
HS10	0.575	0.561	0.598	0.014	0.023
HS11	0.442	0.449	0.547	0.007	0.105
HS12	0.362	0.201	0.571	0.161	0.209
HS13	0.408	0.365	0.557	0.043	0.149
HS14	0.391	0.475	0.337	0.084	0.054
HS15	0.313	0.28	0.422	0.033	0.109
HS16	0.219	0.148	0.429	0.071	0.210
HS17	0.292	0.26	0.416	0.032	0.124
HS18	0.387	0.366	0.497	0.021	0.110
HS19	0.524	0.52	0.479	0.004	0.045
HS20	0.509	0.512	0.561	0.003	0.052
HS21	0.368	0.372	0.473	0.004	0.105
HS22	0.53	0.525	0.517	0.005	0.013

HS23	0.424	0.429	0.499	0.005	0.075
HS24	0.432	0.353	0.471	0.079	0.039
HS25	0.454	0.359	0.476	0.095	0.022
HS26	0.426	0.425	0.468	0.001	0.042
HS27	0.399	0.283	0.47	0.116	0.071
HS28	0.476	0.479	0.498	0.003	0.022
HS29	0.559	0.447	0.481	0.112	0.078
HS30	0.525	0.464	0.503	0.061	0.022
HS31	0.568	0.321	0.556	0.247	0.012
HS32	0.455	0.365	0.405	0.090	0.050
HS33	0.517	0.507	0.429	0.010	0.088
HS34	0.503	0.561	0.502	0.058	0.001
HS35	0.48	0.577	0.431	0.097	0.049
HS36	0.462	0.449	0.53	0.013	0.068
HS37	0.509	0.595	0.518	0.086	0.009
HS38	0.467	0.611	0.478	0.144	0.011
HS39	0.383	0.509	0.389	0.126	0.006
HS40	0.38	0.457	0.423	0.077	0.043
HS41	0.37	0.403	0.342	0.033	0.028
HS42	0.46	0.363	0.524	0.097	0.064
HS43	0.352	0.323	0.432	0.029	0.080
HS44	0.583	0.583	0.544	0.000	0.039
HS45	0.561	0.463	0.529	0.098	0.032
HS46	0.412	0.42	0.449	0.008	0.037
HS47	0.466	0.57	0.487	0.104	0.021
HS48	0.402	0.414	0.528	0.012	0.126

HS49	0.355	0.432	0.456	0.077	0.101
HS50	0.473	0.462	0.543	0.011	0.070
HS51	0.332	0.35	0.462	0.018	0.130
HS52	0.567	0.396	0.532	0.171	0.035
HS53	0.409	0.475	0.393	0.066	0.016
HS54	0.502	0.494	0.537	0.008	0.035
HS55	0.313	0.281	0.409	0.032	0.096
HS56	0.378	0.34	0.438	0.038	0.060
HS57	0.468	0.545	0.452	0.077	0.016
HS58	0.423	0.364	0.55	0.059	0.127
HS59	0.415	0.348	0.369	0.067	0.046
HS60	0.385	0.51	0.32	0.125	0.065
HS61	0.245	0.392	0.246	0.147	0.001
HS62	0.232	0.247	0.387	0.015	0.155
HS63	0.269	0.2	0.399	0.069	0.130
HS64	0.236	0.159	0.428	0.077	0.192
HS65	0.439	0.43	0.506	0.009	0.067
HS66	0.387	0.419	0.436	0.032	0.049
HS67	0.409	0.425	0.421	0.016	0.012
HS68	0.396	0.281	0.583	0.115	0.187
HS69	0.416	0.425	0.504	0.009	0.088
HS70	0.369	0.316	0.459	0.053	0.090
HS71	0.377	0.404	0.421	0.027	0.044
HS72	0.427	0.422	0.467	0.005	0.040
HS73	0.516	0.517	0.469	0.001	0.047
HS74	0.326	0.364	0.349	0.038	0.023

HS75	0.367	0.423	0.442	0.056	0.075
HS76	0.402	0.291	0.513	0.111	0.111
HS77	0.358	0.368	0.416	0.010	0.058
HS78	0.356	0.396	0.407	0.040	0.051
HS79	0.388	0.486	0.447	0.098	0.059
HS80	0.315	0.412	0.317	0.097	0.002
HS81	0.458	0.441	0.483	0.017	0.025
HS82	0.34	0.489	0.337	0.149	0.003
HS83	0.394	0.481	0.375	0.087	0.019
HS84	0.371	0.483	0.364	0.112	0.007
HS85	0.333	0.394	0.305	0.061	0.028
HS86	0.269	0.447	0.273	0.178	0.004
HS87	0.224	0.294	0.261	0.070	0.037
HS88	0.329	0.429	0.284	0.100	0.045
HS89	0.256	0.446	0.234	0.190	0.022
HS90	0.544	0.464	0.521	0.080	0.023
HS91	0.625	0.701	0.578	0.076	0.047
HS92	0.357	0.372	0.479	0.015	0.122
HS93	0.485	0.586	0.474	0.101	0.011
HS94	0.639	0.58	0.586	0.059	0.053
HS95	0.436	0.424	0.499	0.012	0.063
HS96	0.332	0.186	0.49	0.146	0.158
HS97	0.397	0.449	0.479	0.052	0.082
HS98	0.549	0.546	0.499	0.003	0.050
HS99	0.333	0.467	0.281	0.134	0.052
HS100	0.47	0.541	0.421	0.071	0.049

HS101	0.348	0.396	0.408	0.048	0.060
HS102	0.439	0.53	0.413	0.091	0.026
HS103	0.568	0.543	0.423	0.025	0.145
HS104	0.352	0.218	0.542	0.134	0.190
HS105	0.284	0.226	0.432	0.058	0.148
HS106	0.479	0.377	0.5	0.102	0.021
HS107	0.187	0.075	0.386	0.112	0.199
HS108	0.418	0.482	0.453	0.064	0.035
HS109	0.343	0.263	0.43	0.080	0.087
HS110	0.288	0.302	0.398	0.014	0.110
HS111	0.489	0.613	0.452	0.124	0.037
HS112	0.464	0.438	0.526	0.026	0.062
HS113	0.499	0.478	0.639	0.021	0.140
HS114	0.512	0.573	0.451	0.061	0.061
HS115	0.592	0.589	0.576	0.003	0.016
HS116	0.37	0.439	0.42	0.069	0.050
HS117	0.583	0.587	0.549	0.004	0.034
HS118	0.565	0.534	0.585	0.031	0.020
HS119	0.458	0.413	0.557	0.045	0.099
HS120	0.601	0.601	0.659	0.000	0.058
HS121	0.365	0.277	0.522	0.088	0.157
HS122	0.331	0.312	0.462	0.019	0.131
HS123	0.423	0.363	0.47	0.060	0.047
HS124	0.309	0.311	0.329	0.002	0.020
HS125	0.43	0.437	0.378	0.007	0.052
HS126	0.244	0.213	0.386	0.031	0.142

HS127	0.318	0.49	0.369	0.172	0.051
HS128	0.391	0.306	0.456	0.085	0.065
HS129	0.31	0.284	0.39	0.026	0.080
HS130	0.274	0.31	0.314	0.036	0.040
HS131	0.371	0.391	0.423	0.020	0.052
HS132	0.333	0.34	0.391	0.007	0.058
HS133	0.387	0.257	0.516	0.130	0.129
HS134	0.469	0.465	0.454	0.004	0.015
HS135	0.338	0.291	0.441	0.047	0.103
HS136	0.402	0.423	0.457	0.021	0.055
HS137	0.44	0.508	0.363	0.068	0.077
HS138	0.515	0.516	0.533	0.001	0.018
HS139	0.294	0.443	0.264	0.149	0.030
HS140	0.397	0.344	0.431	0.053	0.034
HS141	0.423	0.396	0.47	0.027	0.047
HS142	0.304	0.328	0.402	0.024	0.098
HS143	0.363	0.384	0.468	0.021	0.105
HS144	0.139	0.073	0.301	0.066	0.162
HS145	0.332	0.355	0.464	0.023	0.132
HS146	0.253	0.275	0.344	0.022	0.091
HS147	0.324	0.335	0.368	0.011	0.044
HS148	0.337	0.292	0.41	0.045	0.073
HS149	0.356	0.354	0.435	0.002	0.079
HS150	0.512	0.403	0.423	0.109	0.089
HS151	0.392	0.431	0.414	0.039	0.022
HS152	0.357	0.497	0.307	0.140	0.050

HS153	0.273	0.385	0.246	0.112	0.027
HS154	0.319	0.373	0.378	0.054	0.059
HS155	0.252	0.22	0.392	0.032	0.140
HS156	0.22	0.238	0.324	0.018	0.104
HS157	0.321	0.326	0.39	0.005	0.069
HS158	0.327	0.429	0.373	0.102	0.046
HS159	0.278	0.409	0.263	0.131	0.015
HS160	0.282	0.461	0.271	0.179	0.011
HS161	0.333	0.408	0.291	0.075	0.042
HS162	0.383	0.362	0.359	0.021	0.024
HS163	0.656	0.598	0.579	0.058	0.077
HS164	0.62	0.673	0.533	0.053	0.087
HS165	0.464	0.459	0.497	0.005	0.033
HS166	0.547	0.551	0.551	0.004	0.004
HS167	0.464	0.549	0.445	0.085	0.019
HS168	0.413	0.473	0.443	0.060	0.030
HS169	0.361	0.283	0.44	0.078	0.079
HS170	0.424	0.444	0.502	0.020	0.078
HS171	0.465	0.587	0.422	0.122	0.043
HS172	0.283	0.225	0.388	0.058	0.105
HS173	0.409	0.428	0.422	0.019	0.013
HS174	0.346	0.317	0.464	0.029	0.118
HS175	0.417	0.318	0.462	0.099	0.045
HS176	0.389	0.4	0.439	0.011	0.050
HS177	0.425	0.37	0.503	0.055	0.078
HS178	0.509	0.401	0.473	0.108	0.036

HS179	0.414	0.491	0.356	0.077	0.058
HS180	0.597	0.522	0.549	0.075	0.048
HS181	0.527	0.383	0.563	0.144	0.036
HS182	0.479	0.329	0.527	0.150	0.048
HS183	0.513	0.402	0.571	0.111	0.058
HS184	0.389	0.228	0.543	0.161	0.154
HS185	0.249	0.068	0.496	0.181	0.247
HS186	0.376	0.253	0.522	0.123	0.146
HS187	0.339	0.192	0.546	0.147	0.207
HS188	0.251	0.209	0.414	0.042	0.163
HS189	0.254	0.174	0.421	0.080	0.167
HS190	0.177	0.081	0.388	0.096	0.211
HS191	0.405	0.479	0.51	0.074	0.105
HS192	0.539	0.478	0.715	0.061	0.176
HS193	0.317	0.388	0.305	0.071	0.012
HS194	0.36	0.454	0.423	0.094	0.063
HS195	0.307	0.508	0.258	0.201	0.049
HS196	0.446	0.561	0.351	0.115	0.095
HS197	0.256	0.394	0.299	0.138	0.043
HS198	0.353	0.388	0.314	0.035	0.039
HS199	0.112	0.122	0.253	0.010	0.141
HS200	0.462	0.453	0.473	0.009	0.011
HS201	0.472	0.528	0.534	0.056	0.062
HS202	0.274	0.395	0.352	0.121	0.078
HS203	0.31	0.484	0.285	0.174	0.025
HS204	0.268	0.388	0.299	0.120	0.031

HS205	0.295	0.416	0.312	0.121	0.017
HS206	0.194	0.339	0.226	0.145	0.032
HS207	0.377	0.527	0.372	0.150	0.005
HS208	0.286	0.362	0.32	0.076	0.034
HS209	0.38	0.441	0.336	0.061	0.044
HS210	0.374	0.532	0.38	0.158	0.006
HS211	0.765	0.736	0.548	0.029	0.217
HS212	0.26	0.289	0.318	0.029	0.058
HS213	0.526	0.688	0.484	0.162	0.042
HS214	0.475	0.571	0.433	0.096	0.042
HS215	0.453	0.483	0.416	0.030	0.037
HS216	0.458	0.443	0.433	0.015	0.025
HS217	0.419	0.213	0.58	0.206	0.161
HS218	0.443	0.431	0.475	0.012	0.032
HS219	0.535	0.393	0.468	0.142	0.067
HS220	0.506	0.332	0.634	0.174	0.128
HS221	0.545	0.468	0.561	0.077	0.016
HS222	0.496	0.455	0.436	0.041	0.060
HS223	0.437	0.402	0.543	0.035	0.106
HS224	0.5	0.345	0.652	0.155	0.152
HS225	0.456	0.352	0.609	0.104	0.153
HS226	0.401	0.372	0.51	0.029	0.109
HS227	0.338	0.301	0.477	0.037	0.139
HS228	0.37	0.329	0.511	0.041	0.141
HS229	0.308	0.252	0.435	0.056	0.127
HS230	0.348	0.232	0.524	0.116	0.176

HS231	0.346	0.302	0.487	0.044	0.141
HS232	0.297	0.207	0.483	0.090	0.186
HS233	0.307	0.244	0.472	0.063	0.165
HS234	0.312	0.288	0.46	0.024	0.148
HS235	0.531	0.431	0.593	0.100	0.062
HS236	0.32	0.327	0.351	0.007	0.031
HS237	0.27	0.174	0.462	0.096	0.192
HS238	0.451	0.261	0.598	0.190	0.147
HS239	0.417	0.442	0.481	0.025	0.064
HS240	0.461	0.407	0.502	0.054	0.041
HS241	0.401	0.496	0.426	0.095	0.025
HS242	0.399	0.465	0.438	0.066	0.039
HS243	0.273	0.587	0.219	0.314	0.054
HS244	0.337	0.392	0.438	0.055	0.101
HS245	0.368	0.404	0.448	0.036	0.080
HS246	0.219	0.309	0.257	0.090	0.038
HS247	0.32	0.454	0.379	0.134	0.059
HS248	0.321	0.363	0.365	0.042	0.044
HS249	0.4	0.515	0.359	0.115	0.041
HS250	0.349	0.37	0.427	0.021	0.078
HS251	0.391	0.472	0.433	0.081	0.042
HS252	0.34	0.478	0.267	0.138	0.073
HS253	0.355	0.505	0.335	0.150	0.020
HS254	0.395	0.526	0.387	0.131	0.008
HS255	0.373	0.406	0.417	0.033	0.044
HS256	0.38	0.448	0.43	0.068	0.050

HS257	0.481	0.551	0.45	0.070	0.031
HS258	0.172	0.198	0.293	0.026	0.121
HS259	0.28	0.409	0.326	0.129	0.046
HS260	0.276	0.431	0.264	0.155	0.012
HS261	0.149	0.106	0.311	0.043	0.162
HS262	0.22	0.129	0.405	0.091	0.185
HS263	0.338	0.349	0.402	0.011	0.064
HS264	0.394	0.437	0.399	0.043	0.005
HS265	0.251	0.432	0.212	0.181	0.039
HS266	0.278	0.441	0.276	0.163	0.002
HS267	0.175	0.279	0.229	0.104	0.054
HS268	0.255	0.353	0.284	0.098	0.029
HS269	0.384	0.436	0.395	0.052	0.011
HS270	0.354	0.417	0.423	0.063	0.069
HS271	0.338	0.278	0.457	0.060	0.119
HS272	0.497	0.556	0.494	0.059	0.003
HS273	0.516	0.517	0.515	0.001	0.001
HS274	0.287	0.186	0.462	0.101	0.175
HS275	0.442	0.626	0.432	0.184	0.010
HS276	0.467	0.432	0.514	0.035	0.047
HS277	0.5	0.424	0.499	0.076	0.001
HS278	0.521	0.469	0.525	0.052	0.004
HS279	0.368	0.392	0.423	0.024	0.055
HS280	0.33	0.32	0.326	0.010	0.004
HS281	0.578	0.478	0.633	0.100	0.055
HS282	0.607	0.444	0.615	0.163	0.008

HS283	0.386	0.348	0.475	0.038	0.089
HS284	0.438	0.288	0.581	0.150	0.143
HS285	0.287	0.165	0.472	0.122	0.185
HS286	0.494	0.328	0.607	0.166	0.113
HS287	0.302	0.376	0.36	0.074	0.058
HS288	0.3	0.351	0.318	0.051	0.018
HS289	0.389	0.313	0.528	0.076	0.139
HS290	0.426	0.377	0.541	0.049	0.115
HS291	0.408	0.39	0.483	0.018	0.075
HS292	0.354	0.413	0.438	0.059	0.084
HS293	0.48	0.487	0.589	0.007	0.109
HS294	0.431	0.38	0.535	0.051	0.104
HS295	0.444	0.402	0.553	0.042	0.109
HS296	0.369	0.304	0.494	0.065	0.125
HS297	0.438	0.379	0.499	0.059	0.061
HS298	0.475	0.444	0.555	0.031	0.080
HS299	0.332	0.357	0.384	0.025	0.052
HS300	0.415	0.385	0.489	0.030	0.074
HS301	0.277	0.334	0.258	0.057	0.019
HS302	0.355	0.447	0.358	0.092	0.003
HS303	0.316	0.409	0.271	0.093	0.045
HS304	0.335	0.438	0.255	0.103	0.080
HS305	0.394	0.592	0.359	0.198	0.035
HS306	0.375	0.508	0.31	0.133	0.065
HS307	0.205	0.228	0.28	0.023	0.075
HS308	0.295	0.558	0.246	0.263	0.049

HS309	0.162	0.263	0.245	0.101	0.083
HS310	0.221	0.367	0.217	0.146	0.004
HS311	0.278	0.37	0.333	0.092	0.055
HS312	0.197	0.277	0.262	0.080	0.065
HS313	0.29	0.446	0.264	0.156	0.026
HS314	0.157	0.303	0.218	0.146	0.061
HS315	0.271	0.272	0.379	0.001	0.108
HS316	0.403	0.632	0.314	0.229	0.089
HS317	0.424	0.455	0.402	0.031	0.022
HS318	0.411	0.537	0.361	0.126	0.050
HS319	0.319	0.427	0.206	0.108	0.113
HS320	0.368	0.607	0.231	0.239	0.137
HS321	0.408	0.599	0.366	0.191	0.042
HS322	0.374	0.475	0.351	0.101	0.023
HS323	0.27	0.38	0.189	0.110	0.081
HS324	0.341	0.499	0.25	0.158	0.091
HS325	0.437	0.605	0.377	0.168	0.060
HS326	0.285	0.425	0.273	0.140	0.012
HS327	0.35	0.452	0.368	0.102	0.018
HS328	0.482	0.554	0.41	0.072	0.072
HS329	0.328	0.452	0.325	0.124	0.003
HS330	0.512	0.596	0.434	0.084	0.078
HS331	0.558	0.737	0.378	0.179	0.180
HS332	0.416	0.573	0.36	0.157	0.056
HS333	0.414	0.527	0.393	0.113	0.021
HS334	0.366	0.58	0.287	0.214	0.079

HS335	0.375	0.567	0.306	0.192	0.069
HS336	0.53	0.736	0.457	0.206	0.073
HS337	0.38	0.559	0.3	0.179	0.080
HS338	0.328	0.479	0.272	0.151	0.056
HS339	0.249	0.334	0.234	0.085	0.015
HS340	0.377	0.333	0.423	0.044	0.046
HS341	0.39	0.568	0.348	0.178	0.042
HS342	0.171	0.279	0.262	0.108	0.091
HS343	0.349	0.44	0.325	0.091	0.024
HS344	0.286	0.384	0.272	0.098	0.014
HS345	0.41	0.482	0.38	0.072	0.030
HS346	0.286	0.446	0.266	0.160	0.020
HS347	0.197	0.325	0.209	0.128	0.012
HS348	0.313	0.343	0.403	0.030	0.090
HS349	0.32	0.358	0.293	0.038	0.027
HS350	0.324	0.454	0.287	0.130	0.037
HS351	0.367	0.382	0.377	0.015	0.010
HS352	0.366	0.49	0.295	0.124	0.071
HS353	0.375	0.537	0.33	0.162	0.045
HS354	0.367	0.437	0.334	0.070	0.033
HS355	0.284	0.436	0.239	0.152	0.045
HS356	0.313	0.473	0.292	0.160	0.021
HS357	0.354	0.439	0.358	0.085	0.004
HS358	0.337	0.435	0.306	0.098	0.031
HS359	0.267	0.3	0.308	0.033	0.041
HS360	0.255	0.416	0.217	0.161	0.038

HS361	0.263	0.323	0.228	0.060	0.035
HS362	0.326	0.355	0.404	0.029	0.078
HS363	0.267	0.375	0.192	0.108	0.075
HS364	0.256	0.279	0.25	0.023	0.006
HS365	0.225	0.32	0.235	0.095	0.010
HS366	0.375	0.509	0.292	0.134	0.083
HS367	0.338	0.383	0.233	0.045	0.105
HS368	0.408	0.555	0.337	0.147	0.071
HS369	0.375	0.415	0.349	0.040	0.026
HS370	0.287	0.401	0.274	0.114	0.013
HS371	0.246	0.401	0.262	0.155	0.016
HS372	0.379	0.555	0.324	0.176	0.055
HS373	0.238	0.398	0.226	0.160	0.012
HS374	0.337	0.476	0.312	0.139	0.025
HS375	0.23	0.297	0.27	0.067	0.040
HS376	0.315	0.349	0.301	0.034	0.014
HS377	0.29	0.423	0.217	0.133	0.073
HS378	0.36	0.444	0.299	0.084	0.061
HS379	0.369	0.507	0.308	0.138	0.061
HS380	0.289	0.498	0.298	0.209	0.009
HS381	0.247	0.439	0.199	0.192	0.048
HS382	0.202	0.285	0.227	0.083	0.025
HS383	0.266	0.487	0.166	0.221	0.100
HS384	0.124	0.315	0.16	0.191	0.036
HS385	0.307	0.423	0.284	0.116	0.023
HS386	0.379	0.516	0.364	0.137	0.015

HS387	0.304	0.269	0.386	0.035	0.082
HS388	0.202	0.146	0.302	0.056	0.100
HS389	0.214	0.279	0.293	0.065	0.079
HS390	0.308	0.365	0.37	0.057	0.062
HS391	0.191	0.186	0.277	0.005	0.086
HS392	0.26	0.195	0.383	0.065	0.123
HS393	0.363	0.456	0.383	0.093	0.020
HS394	0.274	0.324	0.31	0.050	0.036
HS395	0.534	0.499	0.523	0.035	0.011
HS396	0.279	0.383	0.253	0.104	0.026
HS397	0.458	0.4	0.376	0.058	0.082
HS398	0.284	0.414	0.308	0.130	0.024
HS399	0.222	0.398	0.236	0.176	0.014
HS400	0.341	0.422	0.286	0.081	0.055
HS401	0.281	0.312	0.297	0.031	0.016
HS402	0.409	0.522	0.412	0.113	0.003
HS403	0.33	0.403	0.352	0.073	0.022
HS404	0.463	0.594	0.447	0.131	0.016
HS405	0.358	0.453	0.351	0.095	0.007
HS406	0.403	0.556	0.327	0.153	0.076
HS407	0.244	0.333	0.303	0.089	0.059
HS408	0.295	0.494	0.232	0.199	0.063
HS409	0.226	0.43	0.186	0.204	0.040
HS410	0.223	0.338	0.249	0.115	0.026
HS411	0.21	0.479	0.131	0.269	0.079
HS412	0.278	0.391	0.257	0.113	0.021

HS413	0.234	0.397	0.245	0.163	0.011
HS414	0.199	0.35	0.199	0.151	0.000
HS415	0.191	0.239	0.225	0.048	0.034
HS416	0.228	0.392	0.204	0.164	0.024
HS417	0.267	0.395	0.281	0.128	0.014
HS418	0.314	0.491	0.287	0.177	0.027
HS419	0.291	0.397	0.268	0.106	0.023
HS420	0.2	0.304	0.241	0.104	0.041
HS421	0.283	0.452	0.219	0.169	0.064
HS422	0.274	0.464	0.28	0.190	0.006
HS423	0.17	0.332	0.209	0.162	0.039
HS424	0.292	0.355	0.318	0.063	0.026
HS425	0.237	0.419	0.229	0.182	0.008
HS426	0.387	0.507	0.26	0.120	0.127
HS427	0.225	0.338	0.217	0.113	0.008
HS428	0.232	0.334	0.29	0.102	0.058
HS429	0.305	0.537	0.276	0.232	0.029
HS430	0.32	0.5	0.257	0.180	0.063
HS431	0.34	0.549	0.288	0.209	0.052
HS432	0.362	0.423	0.298	0.061	0.064
HS433	0.317	0.446	0.321	0.129	0.004
HS434	0.352	0.411	0.276	0.059	0.076
HS435	0.44	0.486	0.426	0.046	0.014
HS436	0.438	0.56	0.409	0.122	0.029
HS437	0.558	0.665	0.395	0.107	0.163
HS438	0.36	0.448	0.298	0.088	0.062

HS439	0.45	0.497	0.356	0.047	0.094
HS440	0.525	0.571	0.375	0.046	0.150
HS441	0.382	0.412	0.328	0.030	0.054
HS442	0.422	0.537	0.266	0.115	0.156
HS443	0.348	0.39	0.264	0.042	0.084
HS444	0.408	0.469	0.267	0.061	0.141
HS445	0.202	0.319	0.197	0.117	0.005
HS446	0.511	0.785	0.348	0.274	0.163
HS447	0.528	0.747	0.339	0.219	0.189
HS448	0.404	0.54	0.345	0.136	0.059
HS449	0.394	0.509	0.257	0.115	0.137
HS450	0.391	0.45	0.296	0.059	0.095
HS451	0.39	0.426	0.332	0.036	0.058
HS452	0.321	0.369	0.357	0.048	0.036
HS453	0.434	0.485	0.398	0.051	0.036
HS454	0.281	0.404	0.233	0.123	0.048
HS455	0.503	0.578	0.448	0.075	0.055
HS456	0.349	0.353	0.389	0.004	0.040
HS457	0.345	0.51	0.176	0.165	0.169
HS458	0.468	0.529	0.417	0.061	0.051
HS459	0.354	0.497	0.281	0.143	0.073
HS460	0.338	0.417	0.238	0.079	0.100
HS461	0.429	0.549	0.32	0.120	0.109
HS462	0.281	0.372	0.237	0.091	0.044
HS463	0.392	0.434	0.364	0.042	0.028
HS464	0.449	0.544	0.412	0.095	0.037

HS465	0.34	0.468	0.27	0.128	0.070
HS466	0.351	0.411	0.312	0.060	0.039
HS467	0.429	0.302	0.337	0.127	0.092
HS468	0.316	0.422	0.215	0.106	0.101
HS469	0.331	0.463	0.227	0.132	0.104
HS470	0.362	0.463	0.24	0.101	0.122
HS471	0.385	0.694	0.189	0.309	0.196
HS472	0.354	0.452	0.238	0.098	0.116
HS473	0.236	0.376	0.18	0.140	0.056
HS474	0.322	0.401	0.228	0.079	0.094
HS475	0.352	0.423	0.26	0.071	0.092
HS476	0.405	0.475	0.218	0.070	0.187
HS477	0.36	0.417	0.269	0.057	0.091
HS478	0.465	0.556	0.356	0.091	0.109
HS479	0.446	0.492	0.376	0.046	0.070
HS480	0.397	0.602	0.291	0.205	0.106
HS481	0.36	0.494	0.279	0.134	0.081
HS482	0.387	0.402	0.345	0.015	0.042
HS483	0.229	0.461	0.179	0.232	0.050
HS484	0.236	0.336	0.279	0.100	0.043
HS485	0.26	0.346	0.288	0.086	0.028
HS486	0.256	0.453	0.268	0.197	0.012
HS487	0.304	0.541	0.271	0.237	0.033
HS488	0.328	0.453	0.24	0.125	0.088
HS489	0.126	0.218	0.191	0.092	0.065
HS490	0.157	0.152	0.262	0.005	0.105

HS491	0.089	0.203	0.156	0.114	0.067
HS492	0.312	0.39	0.193	0.078	0.119
HS493	0.253	0.462	0.218	0.209	0.035
HS494	0.29	0.376	0.243	0.086	0.047
HS495	0.127	0.29	0.178	0.163	0.051
HS496	0.133	0.226	0.233	0.093	0.100
HS497	0.288	0.489	0.21	0.201	0.078
HS498	0.283	0.479	0.178	0.196	0.105
HS499	0.179	0.423	0.17	0.244	0.009
HS500	0.286	0.437	0.26	0.151	0.026
HS501	0.162	0.28	0.165	0.118	0.003
HS502	0.242	0.339	0.286	0.097	0.044
Av				8.89%	6.56%
CV				7.72%	
